# Stokes meta-hologram toward optical cryptography

**DOI:** 10.1038/s41467-022-34542-9

**Published:** 2022-11-05

**Authors:** Xuyue Guo, Peng Li, Jinzhan Zhong, Dandan Wen, Bingyan Wei, Sheng Liu, Shuxia Qi, Jianlin Zhao

**Affiliations:** Key Laboratory of light field manipulation and information acquisition, Ministry of Industry and Information Technology, and Shaanxi Key Laboratory of Optical Information Technology, School of Physical Science and Technology, Northwestern Polytechnical University, Xi’an, 710129 China

**Keywords:** Metamaterials, Optical physics, Photonic devices

## Abstract

Optical cryptography manifests itself a powerful platform for information security, which involves encrypting secret images into visual patterns. Recently, encryption schemes demonstrated on metasurface platform have revolutionized optical cryptography, as the versatile design concept allows for unrestrained creativity. Despite rapid progresses, most efforts focus on the functionalities of cryptography rather than addressing performance issues, such as deep security, information capacity, and reconstruction quality. Here, we develop an optical encryption scheme by integrating visual cryptography with metasurface-assisted pattern masking, referred to as Stokes meta-hologram. Based on spatially structured polarization pattern masking, Stokes meta-hologram allows multichannel vectorial encryption to mask multiple secret images into unrecognizable visual patterns, and retrieve them following Stokes vector analysis. Further, an asymmetric encryption scheme based on Stokes vector rotation transformation is proposed to settle the inherent problem of the need to share the key in symmetric encryption. Our results show that Stokes meta-hologram can achieve optical cryptography with effectively improved security, and thereby paves a promising pathway toward optical and quantum security, optical communications, and anticounterfeiting.

## Abstract (web summary)

Achieving optical cryptography scheme with both high capacity and security is highly desirable. Here, authors report a Stokes meta-hologram with a hierarchical encryption strategy that allows vector encryptions to produce depth-masked ciphertexts.

## Introduction

Over the years, information security has always been of vital importance in communication. Especially in the digital era of contemporary society, securely preserving private information is necessary and urgent to alleviate the concerns about data sharing and data misuse. Diverse cryptographic techniques^1–6^ have been sparked to store information and protect them from attack. Among them, optical cryptography^7–11^ delineates a distinctive and excellent framework to advance such domain forward, with regard to the unique features such as high speed, parallel processing, and abundant degrees of freedom (DoFs, e.g., amplitude, phase, polarization, frequency, and orbital angular momentum)^12^. Generally, optical cryptography involves encrypting secret images into visual patterns (as ciphertext), in which no encrypted information can be directly extracted unless decrypting with specific secret keys. Therefore, the secret information can be highly secured and communicated to the intended recipients and remain invisible to unauthorized users. Nevertheless, the bulky size resulting from increasing cryptographic complexity hinders the application of optical cryptography in compact systems.

The pioneering work that combines metasurface with ghost imaging^13^ attracts considerable attention for optical cryptography and can be achieved through meta-hologram, providing a new framework to solve the integration problem and enhance the security level. Thanks to the unparalleled modulation capability on multiple DoFs^14,15^, metasurface-based optical cryptography^16–21^ has subsequently attracted considerable attention, because of the potential in boosting multi-image^22,23^ and color image encryption^24,25^, as well as multi-dimensional key design^26–28^. In addition, the subwavelength-pixel light field manipulation enables metasurface dense data processing. So far, the metasurface-based encryption frameworks are commonly concentrating on the expansion of encryption capacity, that is, using innovative vectorial-^29,30^, multichromatic-^31,32^, and OAM (orbital angular momentum)-holograms^33–35^ to exploit encrypting channels. For instance, encrypting secret images (attached on phase^36^, amplitude^37^ or complex amplitude^38^ of light fields) into independent polarization channels (orthogonal polarization pairs^36^, nonorthogonal polarization pairs^39,40^, or arbitrary polarization combination^41–43^), or hiding images in nonuniform polarization distribution by exploiting Malus’ law^44,45^ and its orientation degeneracy^46,47^. Despite the tremendous developments in this emerging platform in past years, the security issues have not been well addressed. The above multichannel encryption schemes are vulnerable to brute force attacks (e.g., undifferentiated polarization analysis), leaving hidden risks for information security. Zheng et al. recently demonstrated a high-security encryption scheme by integrating metasurface imaging and computational imaging^48^, whereas the information capacity is related to and limited by the shift matrix.

**Fig. 1:**
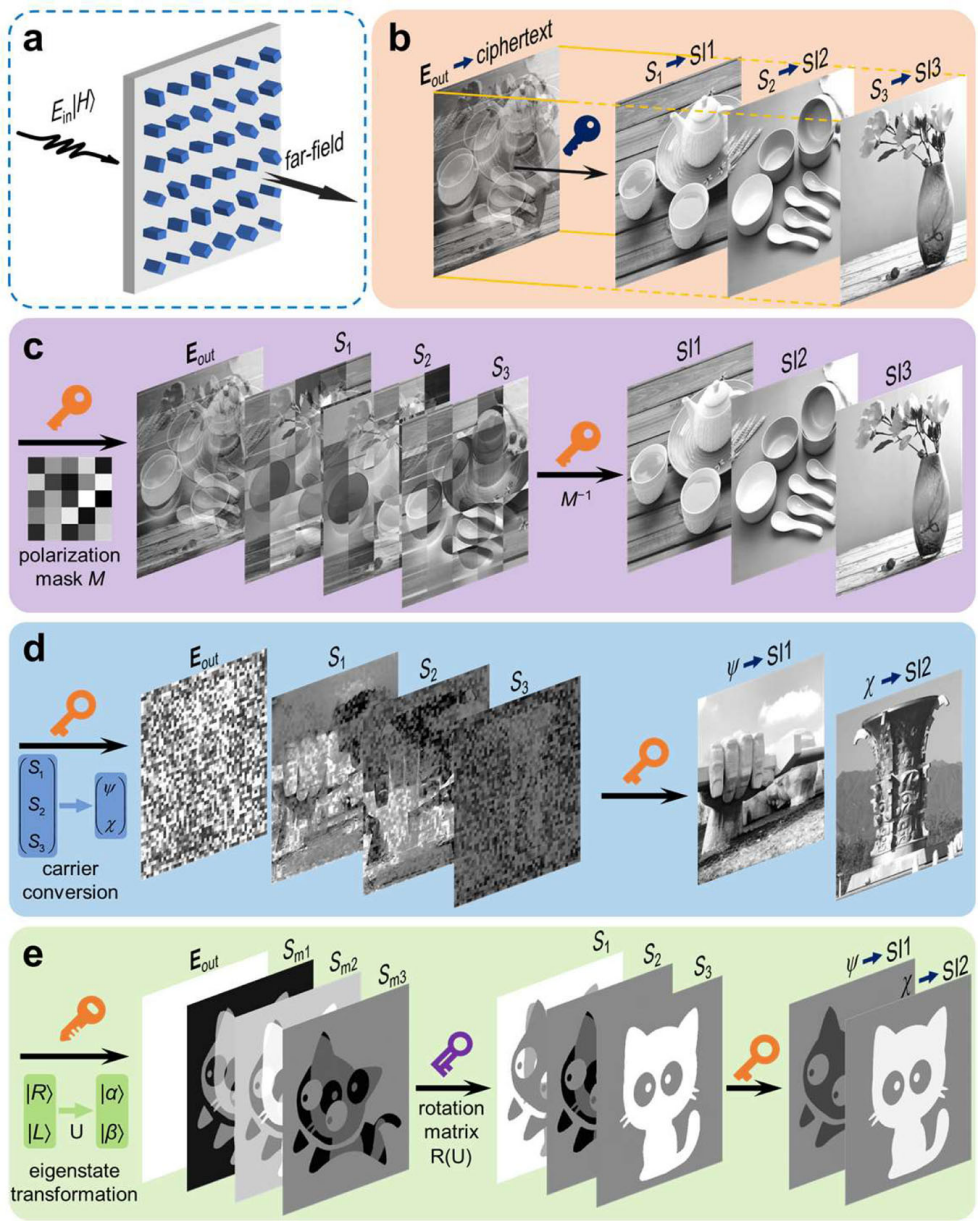
Conceptual illustration of Stokes meta-hologram. **a**, **b** Fundamental Stokes vector encryption. The meta-hologram generates a fully-polarized far-field **E**_out_ (as ciphertext) encrypted with secret images (SI) in Stokes vector, i.e., (SI1, SI2, SI3)^T^ = (*S*_1_, *S*_2_, *S*_3_)^T^, when it is illuminated with a uniform light beam of |*H*〉 polarization state. **c** Mueller matrix encryption. A pixeled polarization mask (depicted as Mueller matrix *M*) is used to perform the second-level encryption, in which the Stokes distribution will be unrecognizable, and a specific secret key is required to decrypt secret images. **d** Angular vector encryption. To eliminate the visible pattern in ciphertext, the vector consisting of azimuth (*ψ*) and elliptical (*χ*) angles on the Poincaré sphere is used as extended secret keys for further-level encryption. The two images are photographs of architecture on the campus of Northwestern Polytechnical University and are used with the permission of Northwestern Polytechnical University. **e** Asymmetric encryption. A rotation transformation of the Stokes vector corresponding to the eigenstate transformation (public key) is introduced as a private key for asymmetric encryption.

In this work, we revisit the capability of metasurface to construct fully-polarized structured light fields and develop a Stokes meta-hologram based on vectorial encryption toward both the security and capacity of optical cryptography. We provide complete design rules on how to design a Stokes meta-hologram with different security levels, of which the principle is illustrated in Fig. 1. The fundamental-level encryption, namely, vectorial encryption based on Stokes parameters, generates a fully-polarized structured light field, as visual ciphertext, in which three secret images are masked into Stokes vector **S** = (*S*_1_, *S*_2_, *S*_3_)^T^. In this scheme, decrypting the ciphertext according to the measured polarization component patterns via the Stokes method can directly retrieve the secret images (Fig. 1b). However, this primary masked ciphertext is easy to be cracked since the attacker can obtain multiple images through polarization analysis and perform further operations according to the visible pattern in the ciphertext. Therefore, a pixeled polarization mask is introduced as the secret key by Mueller matrix transformation (Fig. 1c). Double polarization pattern masking improves the security level and the robustness of ciphertext, while in which, the visible pattern is yet prone to leak some information. In this respect, an information carrier conversion based on the Poincaré sphere angular vector is then introduced in the polarization pattern masking process (Fig. 1d). Owing to the multiple encryption processes and more DoFs, visible pattern in the ciphertext can be effectively eliminated. In order to further improve the encryption level, Stokes vector rotation transformation R(U), as a private key, is introduced to break the symmetry relationship of the secret keys in encrypting and decrypting processes (Fig. 1e). The asymmetric scheme can isolate an encrypted image from the mask (public key) holders, creating extreme improvement in information security.

## Results

### Stokes meta-hologram

As shown in Fig. 1a, the Stokes meta-hologram is desired to yield the ciphertext in the far-field under the illumination of an incident beam with uniform amplitude (*E*_in_ = 1) and definite polarization, e.g., the horizontal polarization |*H*〉. To construct fully-polarized information **E**_out_, the amplitude, phase, and polarization of the transmitted light beam are supposed to experience a completely decoupled modulation through the meta-hologram. Meanwhile, suitable multiplexing methods are as well necessary in favor of the improvement of information capacity. Consequently, we design a dual-channel meta-hologram in which the amplitude, phase, and polarization can be simultaneously and independently modulated on each spatial channel.

**Fig. 2:**
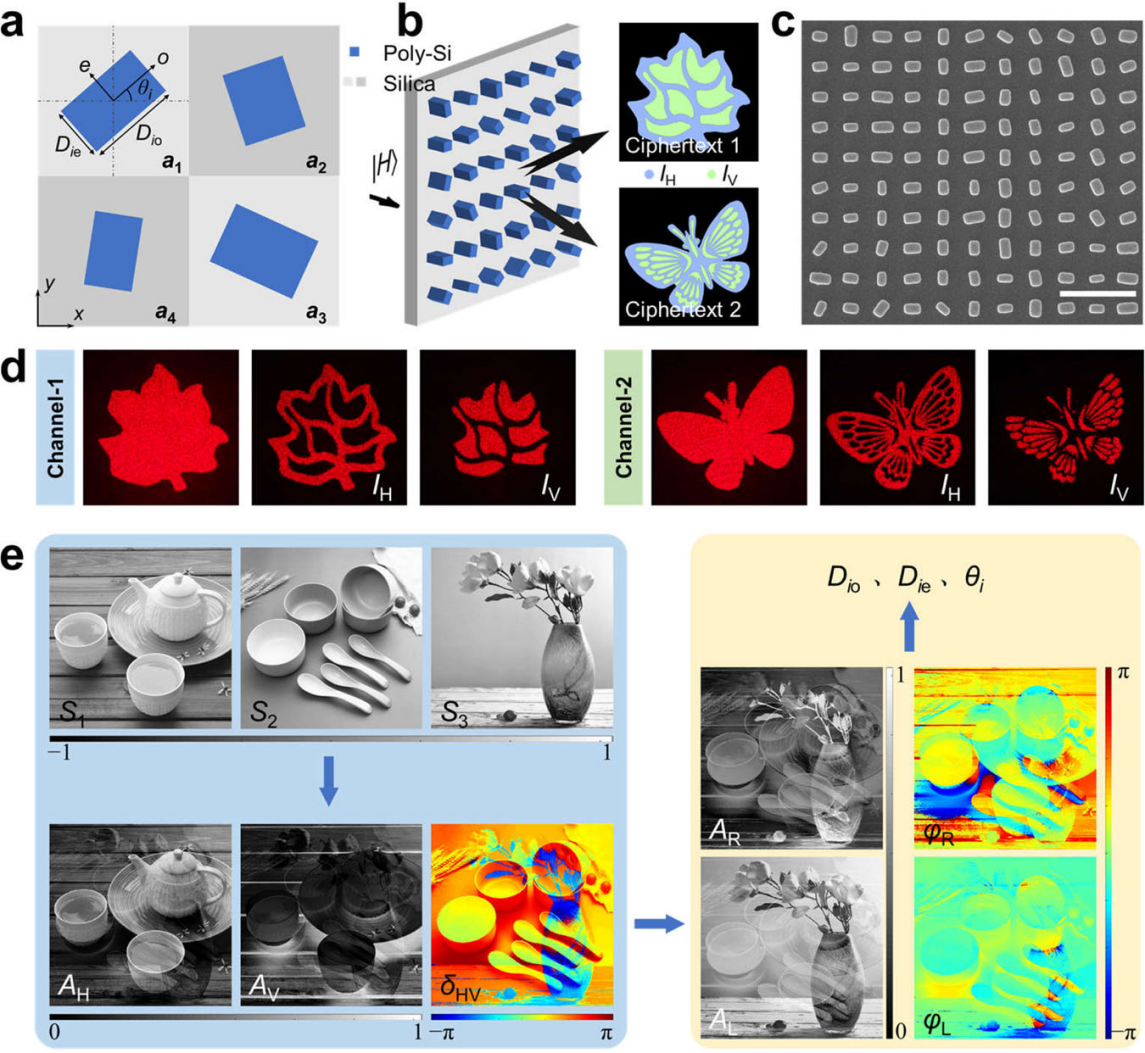
Design principle of Stokes meta-hologram. **a** Schematic illustration of the tetratomic macro-pixel unit cell, which comprises four types of rectangular nanopillars denoted as meta-atoms *a*_1_, *a*_2_, *a*_3_, and *a*_4_ with variant geometrical sizes *D*_*i*o_ and *D*_*i*e_, and orientation angles *θ*_*i*_, (*i* = *a*_1_, *a*_2_, *a*_3_, *a*_4_). The period and height of the macro-pixel unit cell are 900 and 550 nm, respectively. **b** Working principle of dual-channel meta-hologram. **c** Scanning electron microscope image of the metasurface. The scale bar is 1 μm. **d** Experiment results of the dual-channel polarization-switchable holographic display to testify the vectorial encryption performance of the designed meta-hologram. **e** Designing process of Stokes meta-hologram for masking three arbitrary image information into Stokes vector (*S*_1_, *S*_2_, *S*_3_)^T^.

The metasurface is composed of birefringent dielectric nanopillars with a tetratomic macro-pixel arrangement, of which each meta-atom behaves as a half-wave plate and has varying geometrical size and rotation angle, i.e., *D*_*i*o_, *D*_*i*e_, and *θ*_*i*_, (*i* = *a*_1_, *a*_2_, *a*_3_, *a*_4_), as shown in Fig. 2a. By skillfully tailoring the polarization-dependent interference^49^, the far-field distributions in two completely decoupled channels can be achieved as

1 $$\begin{array}{c}{{{{{{\bf{E}}}}}}}_{{{{{{\rm{out}}}}}}}^{1}={ {\mathcal F} }\{2\,\cos ({\xi }_{11})\exp ({{{{{\rm{i}}}}}}{\phi }_{11})|R\rangle+2\,\cos ({\xi }_{12})\exp ({{{{{\rm{i}}}}}}{\phi }_{12})|L\rangle \}\\ \,\,\,\,{{{{{{\bf{E}}}}}}}_{{{{{{\rm{out}}}}}}}^{2}={ {\mathcal F} }\{2\,\cos ({\xi }_{21})\exp ({{{{{\rm{i}}}}}}{\phi }_{21})|R\rangle+2\,\cos ({\xi }_{22})\exp ({{{{{\rm{i}}}}}}{\phi }_{22})|L\rangle \},\end{array}$$

where, |*R*〉 and |*L*〉 represent the right- (RCP) and left-hand circularly polarized (LCP) bases, and *ξ*_11_, *ξ*_12_, *ξ*_21_, *ξ*_22_, *ϕ*_11_, *ϕ*_12_, *ϕ*_21_, *ϕ*_22_ are preestablished phase patterns. The detailed derivation is shown in Supplementary Note 1. A dual-channel Malus meta-hologram is first implemented to testify the performance, as shown in Fig. 2b. Two sets of complementary images are separately encrypted into the horizontal |*H*〉 and vertical |*V*〉 states on each channel, i.e., four independent images are encrypted into a meta-hologram. The scanning electron microscope image and experiment results of the meta-hologram are shown in Fig. 2c, d, respectively. Under linear polarization incidence, each channel displays a uniform holographic pattern composed of |*H*〉 and |*V*〉 polarization multiplexed images, which can be observed by changing the orientation of the polarization analyzer.

We further design the Stokes meta-hologram by using this dual-channel configuration, to create spatial polarization pattern masked ciphertexts with high capacity. The fundamental-level encryption, that is, masking three arbitrary images into Stokes vector **S** = (*S*_1_, *S*_2_, *S*_3_)^T^, is illustrated in Fig. 2e. Firstly, the secret images (SI1, SI2, and SI3) are mapped to *S*_1_, *S*_2_, and *S*_3_ through a linear transformation (2*X*/255 − 1), where *X* represents the original grayscale value. Sophisticated transformation methods, such as nonlinear transformation and matrix shift operation^48^, can be used for high-security encryption. Second, according to the Stokes vector, we retrieve the Jones vector, with linearly polarized basic states:

2 $$	{S}_{1}={A}_{{{{{{\rm{H}}}}}}}^{2}-{A}_{{{{{{\rm{V}}}}}}}^{2}\\ 	{S}_{2}=2{A}_{{{{{{\rm{H}}}}}}}{A}_{{{{{{\rm{V}}}}}}}\,\cos ({\delta }_{{{{{{\rm{HV}}}}}}})\\ 	{S}_{3}=2{A}_{{{{{{\rm{H}}}}}}}{A}_{{{{{{\rm{V}}}}}}}\,\sin ({\delta }_{{{{{{\rm{HV}}}}}}}).$$

Therefore, the desired fully-polarized field is decomposed to the amplitude (*A*_H_, *A*_V_) and phase difference (*δ*_HV_) of the |*H*〉 and |*V*〉 components and then transformed into the complex amplitudes of RCP and LCP. Then, the preestablished phase patterns are generated according to Supplementary Eqs. (8–10), and the required phase responses and orientation angles (*θ*_*i*_) can be obtained from Supplementary Eq. (6). Finally, matching geometries (*D*_*i*o_, *D*_*i*e_) are selected to map the meta-hologram according to phase responses. In the decryption of each channel, the secret images masked in the Stokes vector can be obtained as

3 $$\begin{array}{c}{S}_{1}={|\langle H|{{{{{{\bf{E}}}}}}}_{{{{{{\rm{out}}}}}}}|}^{2}-{|\langle V|{{{{{{\bf{E}}}}}}}_{{{{{{\rm{out}}}}}}}|}^{2}\hfill\\ {S}_{2}={|\langle A|{{{{{{\bf{E}}}}}}}_{{{{{{\rm{out}}}}}}}|}^{2}-{|\langle D|{{{{{{\bf{E}}}}}}}_{{{{{{\rm{out}}}}}}}|}^{2}\hfill\\ {S}_{3}={|\langle R|{{{{{{\bf{E}}}}}}}_{{{{{{\rm{out}}}}}}}|}^{2}-{|\langle L|{{{{{{\bf{E}}}}}}}_{{{{{{\rm{out}}}}}}}|}^{2}.\hfill\end{array}$$

Where, |*A*〉 and |*D*〉 correspond to the antidiagonal and diagonal polarization states, respectively.

### Mueller matrix encryption

A two-dimensional polarization mask is a common selection to enhance the security of ciphertext in Stokes vector encryption, whose encrypting process can be described by the pixeled Mueller matrix as

4 $$\left(\begin{array}{c}{S}_{0}^{{{{{{\rm{out}}}}}}}\\ {S}_{1}^{{{{{{\rm{out}}}}}}}\\ {S}_{2}^{{{{{{\rm{out}}}}}}}\\ {S}_{3}^{{{{{{\rm{out}}}}}}}\end{array}\right)={M}_{{{{{{\rm{n}}}}}}}(\theta )\cdot \cdot \cdot {M}_{1}(\theta )\left(\begin{array}{c}{S}_{0}^{{{{{{\rm{in}}}}}}}\\ {S}_{1}^{{{{{{\rm{in}}}}}}}\\ {S}_{2}^{{{{{{\rm{in}}}}}}}\\ {S}_{3}^{{{{{{\rm{in}}}}}}}\end{array}\right),$$

where, *M*(*θ*) represents the 4 × 4 Mueller matrix with spatially varying modulation, which can be a polarizer, a phase retarder, and so on. One can regard the cascaded modulation effect *M*_1_(*θ*)⋅⋅⋅*M*_*n*_(*θ*) as the secret key, or create multiple secret keys by separating the modulation effect and cascade order. Both the modulation effect and the cascade order are unlimited, therefore, the possibility of the attacker decrypting an unknown image is thus strongly reduced^50^.

**Fig. 3:**
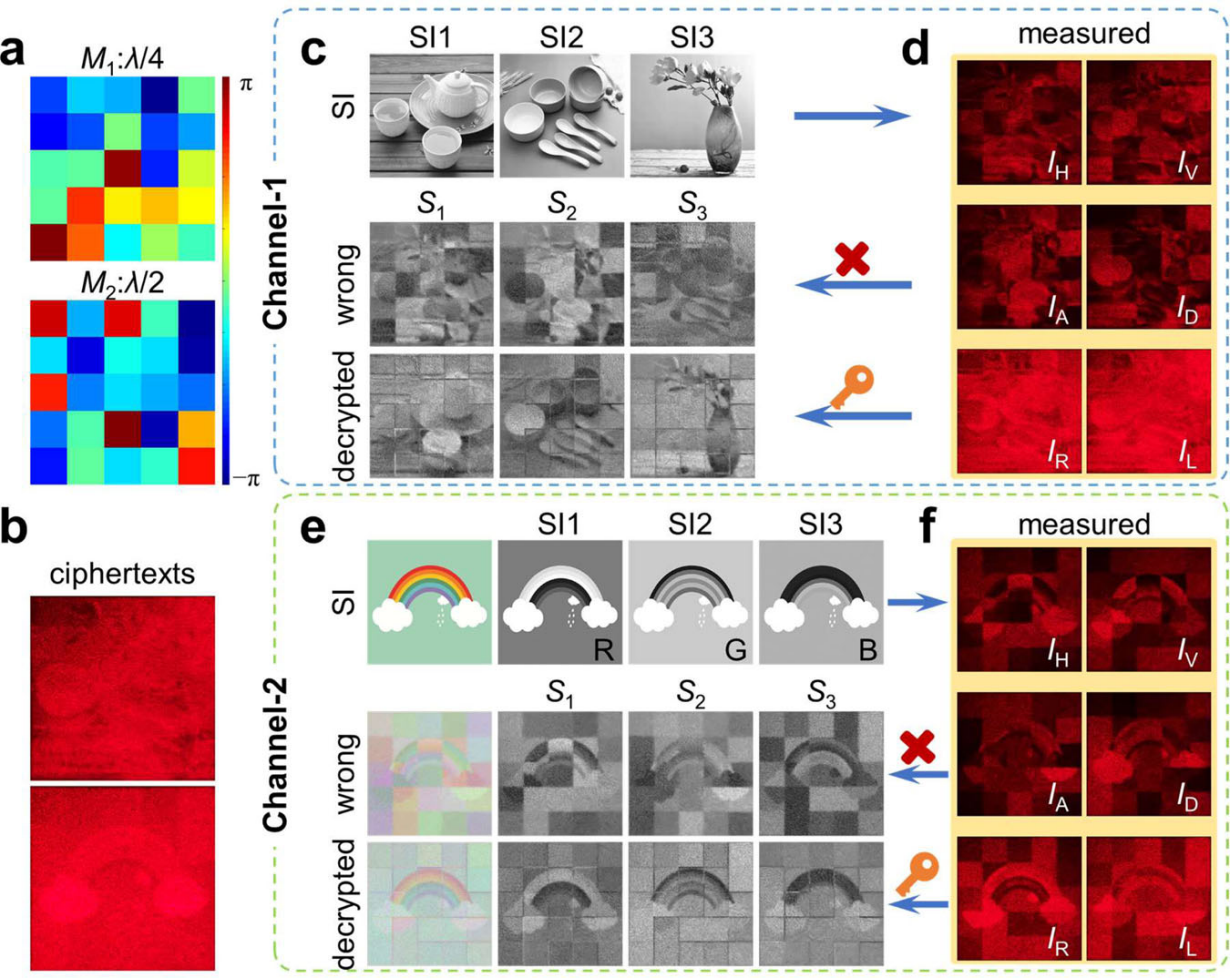
Mueller matrix encryption. **a** Random direction in Mueller matrices of pixelated wave plates. **b** Measured ciphertext (total intensity) patterns. **c**–**f** Decrypted results with and without the secret key. Three grayscale images and an RGB color image are encrypted into Stokes vectors (SI1, SI2, SI3)^T^ (first rows of **c** and **e**) and then transformed into vectorial ciphertexts after a pixelated polarization mask, which is equivalent to the cascaded Mueller matrices *M*_2_*M*_1_. For this two-level vectorial encryption, any measured polarization components (**d** and **f**) and the directly decrypted results by Stokes vector (*S*_1_, *S*_2_, *S*_3_)^T^ (second rows of **c** and **e**) can not present secret images. Whereas, through decryption with a secret key, the secret images can be extracted accurately (third rows of **c** and **e**).

In the experimental demonstration, we exhibit two types of encryptions in two channels, e.g., simultaneously encoding arbitrary three grayscale images and an RGB color image into the Stokes meta-hologram, i.e., up to six images can be encrypted at the same time. The Mueller matrices of cascaded quarter- and half-wave plates with random directions (Fig. 3a) both have 5 × 5 pixels (the security level can be further enhanced with more pixels, which is shown in Supplementary Fig. 15). Compared with the fundamental-level ones (Supplementary Fig. 1), the dual-channel ciphertext patterns (Fig. 3b), measured intensity patterns of different polarization components (Fig. 3d, f), as well as the directly Stokes vector decrypted results (second rows of Fig. 3c, e) all present chaotic information. For accurate decryption, a secret key corresponding to these cascaded Mueller matrices is required to reorder the chaotic distributions, as shown in the third rows of Fig. 3c, e. Obviously, the secret images are stored intactly, and cannot be directly obtained from any single polarization measurement. The experiment and simulation details are shown in Supplementary Figs. 3,4, respectively. Since the captured images are slightly distorted due to the far-field projection, a misalignment of the pixelated polarization mask will be introduced when decrypting. The slight distortion in decrypted images is due to the imperfect fabrication of metasurfaces, and intensity measurement error caused by off-axis aberration and manual capture.

**Fig. 4:**
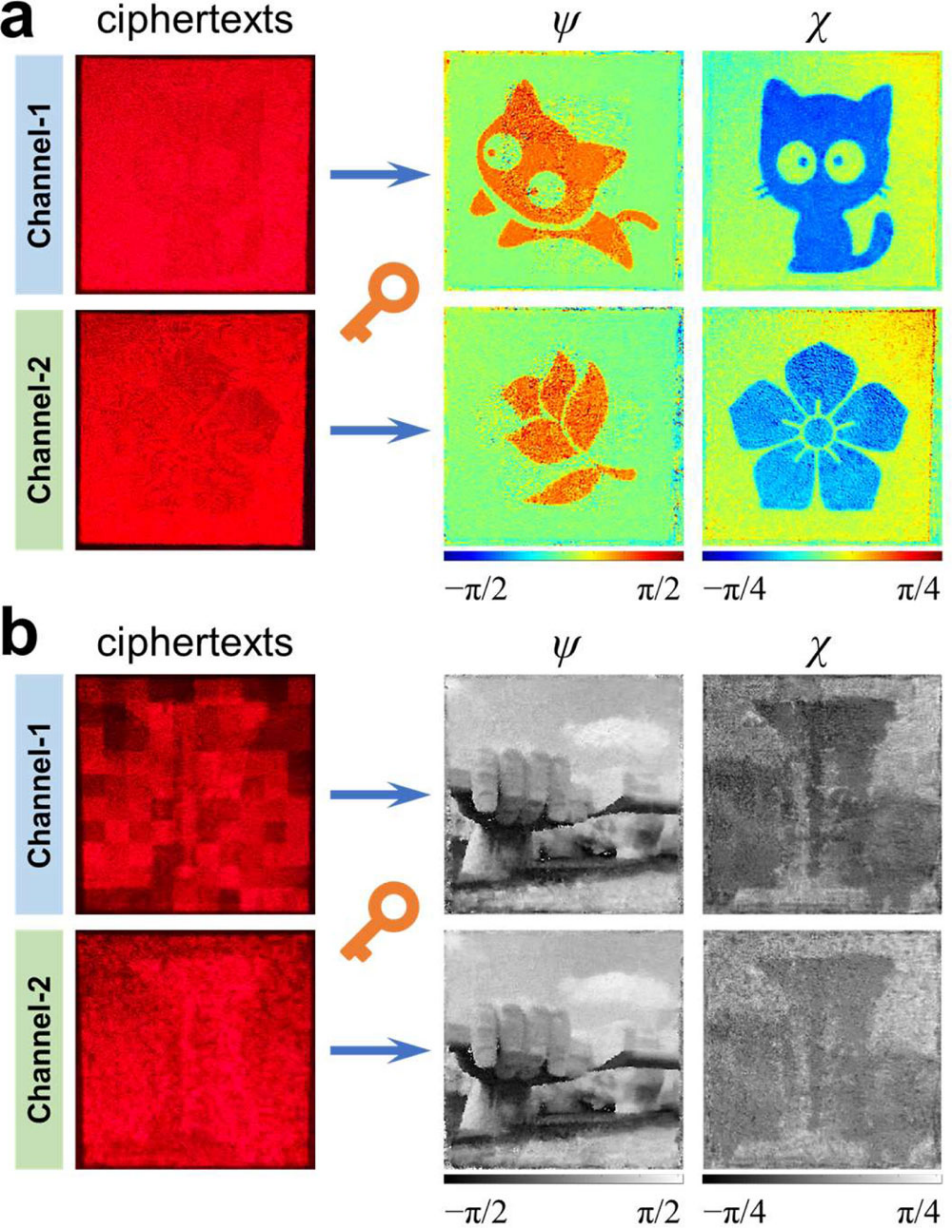
Angular vector encryption on Poincaré sphere. **a**, **b** Uniform and ununiform ciphertext (total intensity *I*_T_) encryptions. In **a**, two secret images are decrypted by characterizing an angular vector (*ψ*, *χ*) on the Poincaré sphere. In contrast, in **b**, two random intensity patterns (10 × 10 pixels for channel-1 and 50 × 50 pixels for channel-2) are used to improve the confidentiality of the measurement of circularly polarized components. The two images are photographs of architecture on the campus of Northwestern Polytechnical University and are used with permission of Northwestern Polytechnical University.

### Angular vector encryption on Poincaré sphere

The above scheme can mask multiple secret images into the Stokes vector. However, the released *S*_0_ results in that the generated ciphertext (total intensity) still presents some plaintext (secret images) information, namely, visible pattern (see the total intensities in Fig. 3b and Supplementary Fig. 1b, e). Despite no secret images can be directly extracted, this scheme still leaves some safety concerns. To address this risk, i.e., to realize a further level of secured encryption, the information carrier can be extended to an angular vector on the Poincaré sphere, which is characterized by the azimuthal and elliptical angles, due to the inherent relationship between the spherical coordinates (namely angular vector) on Poincaré sphere and the Stokes vector in Cartesian coordinates:

5 $$\tan 2\psi={{S}}_{2}/{S}_{1}\\ \sin 2\chi={S}_{3}/{S}_{0}.$$

Therefore, by sacrificing some capacity, the security of ciphertext can be enhanced. Figure 4a shows an experimental verification, for clarity, no Mueller matrix is introduced (simulation results with the Mueller matrix are shown in Supplementary Fig. 16). Clearly, a uniform ciphertext is observed (left panel), but two secret images can be decrypted when characterizing the spatially structured polarization pattern in the azimuth and elliptical angle (right panel).

Theoretically, under the circular polarization eigenstates, the ellipticity only depends on the amplitude ratio of the RCP and LCP states. Therefore, when the ciphertext has a uniform intensity profile, the secret image encrypted on the elliptical angle can be directly observed by merely measuring the RCP or LCP component (see the *I*_R_ and *I*_L_ shown in Supplementary Fig. 5b, d). To eliminate such security risk, we introduce a random intensity distribution to the circular polarization eigenstates with a guaranteed amplitude ratio. Two photographs of architecture on our campus are chosen as secret images. The experiment results are shown in Fig. 4b, and the random intensity patterns are 10 × 10 (upper) and 50 × 50 (lower) pixels in different channels, respectively (details are shown in Supplementary Fig. 6). Clearly, random intensities attached on the LCP dilute the information that can be directly observed. The noise in the decrypted image on an elliptical angle comes from the measurement error of the random intensity distribution. It is worth noting that, the secret images encrypted on azimuth associated with phase can always be recovered well (see decrypted *ψ* in Fig. 4b), whereas, errors in intensity measurement result in some unevenly distributed grids in elliptical angle decryption (see decrypted *χ* in Fig. 4b), which corresponds to the lower values of random intensity. These errors can be averaged by increasing the pixel number of the random intensity pattern, or the secret image can be set to the same pixel number as the random intensity pattern to avoid the loss of grayscale in a continuous gray image, the corresponding demonstration is shown in Supplementary Fig. 7.

### Asymmetric encryption

The above presentations all belong to symmetric encryption, in which the secret key works in both encryption and decryption processes. Whereas, asymmetric encryption uses two keys to boost security, i.e., the public key for encryption and the private key for decryption, eliminating the need to share the key. To break such a symmetry relationship, we introduce an eigenstate transformation on the Poincaré sphere to reconstruct the Stokes vector.

**Fig. 5:**
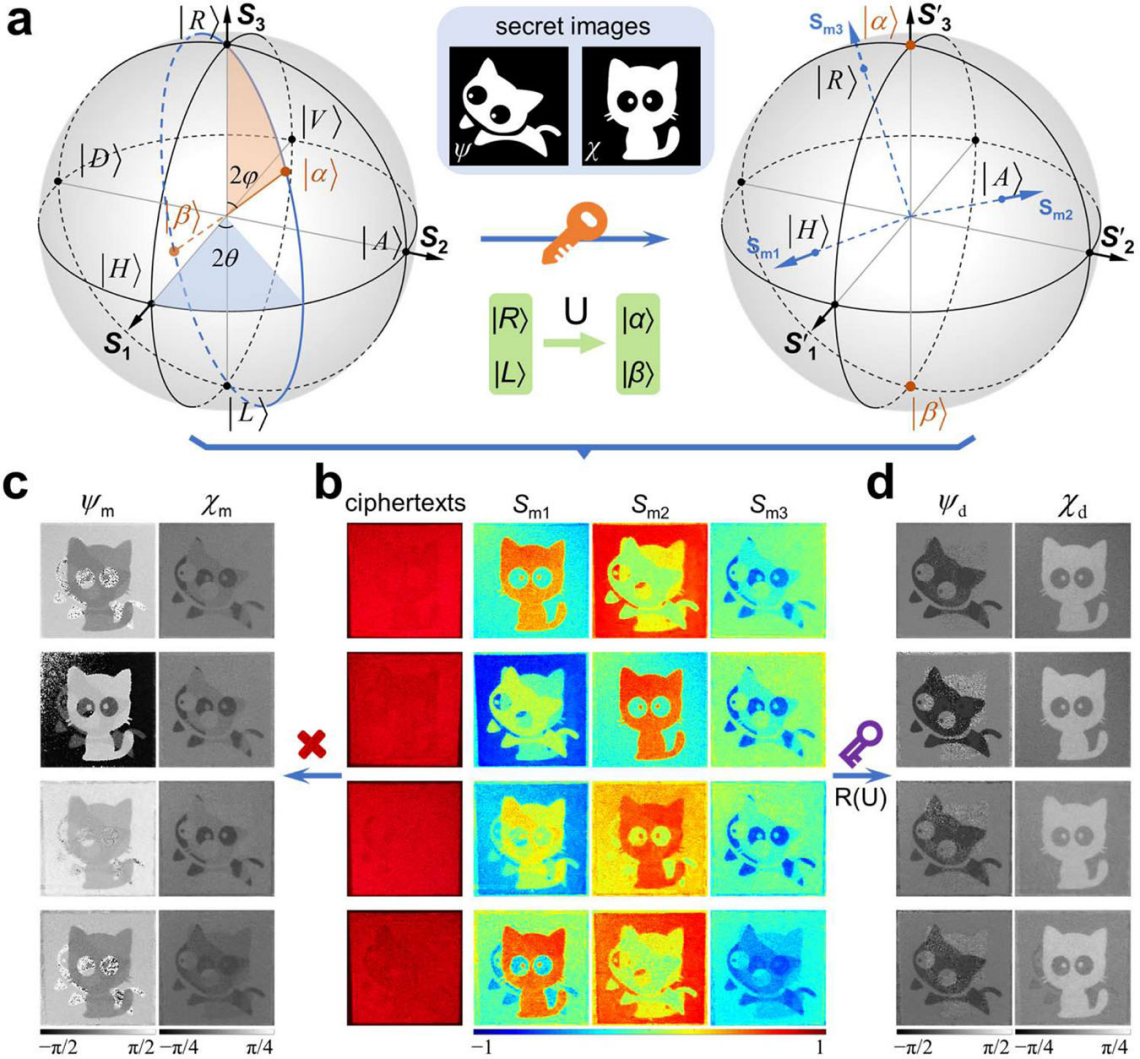
Asymmetric encryption scheme. **a** Rotation transformation of the Poincaré sphere corresponding to the transformation of eigenstates from originally circular polarizations to an arbitrary eigenstate pair |*α*〉 and |*β*〉. Inset (upper): The secret images are encrypted in azimuth and elliptical angles. Inset (bottom): Transformation of eigenstates. **b** Measured ciphertexts and Stokes vectors (*S*_m1_, *S*_m2_, *S*_m3_). **c**, **d** Decrypted images from measured information without and with the private key. The results shown in the first to fourth rows of **b**–**d** correspond to the Poincaré sphere with different eigenstates: |*H*〉-|*V*〉, |*A*〉-|*D*〉, |*α*_1_〉-|*β*_1_〉, |*α*_2_〉-|*β*_2_〉, and the coordinates of |*α*_1_〉 and |*α*_2_〉 are (π/3, π/2) and (0, π/3), respectively.

On a normal Poincaré sphere, any polarization state |*α*〉 can be characterized by a special angular vector (2*ψ*, 2*χ*)^T^ with respect to two eigenstates of RCP and LCP (i.e., the north and south poles) as

6 $$|\alpha \rangle=\,\cos (\varphi )\exp ({{{{{\rm{i}}}}}}\theta )|R\rangle+\,\sin (\varphi )\exp (-{{{{{\rm{i}}}}}}\theta )|L\rangle .$$

where, *φ* and *θ* represent spherical coordinates. Its orthogonal polarization state |*β*〉, located on the sphere that is symmetrical about the origin, as shown in the left panel of Fig. 5a. In our asymmetric encryption scheme, the secret images (middle panel of Fig. 5a) are still encrypted into the angular vector, but an eigenstate transformation is introduced to break the symmetry, that is, implementing the complex amplitude distribution on the original circular polarization eigenstates (in Supplementary Eq. (1)) to any other pair of orthogonal eigenstates (as public key), as

7 $${A}_{R}\exp ({{{{{\rm{i}}}}}}{\varphi }_{R})|\alpha \rangle+{A}_{{{{{{\rm{L}}}}}}}\exp ({{{{{\rm{i}}}}}}{\varphi }_{{{{{{\rm{L}}}}}}})|\beta \rangle,$$

where, *A*_R_exp(i*φ*_R_) and *A*_L_exp(i*φ*_L_) are the complex amplitudes from decomposing the desired fully-polarized field under circular polarization eigenstates. After such transformation, the spatially structured polarization of the field will be redefined. The right panel in Fig. 5a shows the Poincaré sphere constructed by the |*α*〉 and |*β*〉 eigenstates, in which all the coordinates of original polarization states are changed, that is, the angular vector of any polarization state rotates a special angle associated with the angular vector of the |*α*〉 state on the normal Poincaré sphere. Whereas, in practice, the measured Stokes vector is still conventionally determined in terms of six polarization components |*H*〉, |*V*〉, |*A*〉, |*D*〉, |*R*〉, |*L*〉, which results in that the measured Stokes vector **S**_m_ = (*S*_m1_, *S*_m2_, *S*_m3_)^T^ do not coincide with the transformed Stokes vector **S**′ = (*S*′_1_, *S*′_2_, *S*′_3_)^T^ and redefined axes. Because the Stokes vector is equivalent to the angular vector, these secret images cannot be decrypted.

To decrypt, the relationship between eigenstate and Stokes vector transformation can be interpreted by the homomorphic correspondence between SU(2) and SO(3) groups in group theory^51^. The eigenstate transformation can be expressed by the Jones matrix as (*α*, *β*)^T^ = U(*R*, *L*)^T^, obviously, U∈SU(2). Meanwhile, the transformation variation of the Stokes vector can be expressed as the modulation of a Mueller matrix **S**′ = *M*_st_**S**. Noting that, since only variation in polarization is involved and the total intensity remains the same, *S*_0_ needs no consideration. Therefore, the transformation of the Mueller matrix is equivalent to the rotation of the Stokes vector by a certain angle, i.e., **S**′ = R(U)**S**, where 3 × 3 matrix R(U)∈SO(3) represents the three-dimensional rotation matrix. According to the homomorphic correspondence between SU(2) and SO(3), the rotation matrix can be derived as^52,53^

8 $${{{{{\rm{R}}}}}}{({{{{{\rm{U}}}}}})}_{ij}=\frac{1}{2}{{{{{\rm{Tr}}}}}}({{\sigma }}_{i}{{{{{\rm{U}}}}}}{\sigma }_{j}{{{{{{\rm{U}}}}}}}^{{{\dagger}} })\,(i,\, j=1,\, 2,\, 3),$$

where, Tr() is the trace of matrix, *σ*_*i*/*j*_ is Pauli spin matrix, and † stands for the Hermitian conjugate. Further, considering the special symmetry of the Poincaré sphere, the above relationship can be regarded as the private key in the decryption process to extract the secret images masked in the original Stokes vector.

In the experimental demonstration, we select |*H*〉-|*V*〉 and |*A*〉-|*D*〉 eigenstates as the public keys to perform the asymmetric encryption, the transformation relationships are shown in Supplementary Fig. 8. According to these relationships, the rotation matrix can be obtained as

9 $${{{{{{\rm{R}}}}}}}_{{{{{{\rm{HV}}}}}}}=\left(\begin{array}{ccc}0 & 1 & 0\\ 0 & 0 & 1\\ 1 & 0 & 0\end{array}\right),\, {{{{{{\rm{R}}}}}}}_{{{{{{\rm{AD}}}}}}}=\left(\begin{array}{ccc}-1 & 0 & 0\\ 0 & 0 & 1\\ 0 & 1 & 0\end{array}\right).$$

The measured Stokes vector (*S*_m1_, *S*_m2_, *S*_m3_)^T^ and the angular vector (*ψ*_m_, *χ*_m_)^T^ directly calculated without private key are shown in the first two rows of Fig. 5b, c. Obviously, the encrypted information cannot be directly obtained from *ψ*_m_ and *χ*_m_. By performing the asymmetric decryption process in Fig. 1e, the secret images can be ultimately extracted from the angle vector (*ψ*_d_, *χ*_d_)^T^, as shown in the first two rows of Fig. 5d. Further, experimental demonstrations based on the Poincaré sphere under a pair of linear polarizations on equator line and elliptical polarizations on zero meridian eigenstates are performed, as shown in the last two rows of Fig. 5b–d. Simulation results are shown in Supplementary Figs. 9, 10.

## Discussion

It is foreseeable that with the speedy development and multifaceted applications of advanced technologies, such as big data, artificial intelligence (AI), cloud computing, and the like, information security will always be an important challenge. To eliminate the threat of information theft and misuse, it is relatively easier to find attackers in the optical domain rather than on the digital internet. Therefore, developing novel optical cryptographic techniques are highly desirable to meet the challenges in the information security domain.

Security and capacity are timeless goals in optical cryptography, but here’s a caveat that most efforts focus on the functionalities rather than fundamental performance. In our proposal, multi-dimensional and multichannel optical encryption is realized by manipulating the light field with multiple DoFs, and an asymmetric encryption scheme is exploited, in which both security and capacity are well addressed. As proofs of principle, ciphertext-only attack (COA)^54,55^, and known-plaintext attack (KPA)^56,57^ are employed to evaluate the security performance (detailed in Supplementary Note 7), of which the results show that the Stokes meta-hologram can effectively avoid brute force attack and the security level can be increasingly improved by polarization mask, carrier conversion, and eigenstate transformation.

In addition, the reconstruction quality is another crucial factor in terms such as storage and encryption of biological information (e.g., fingerprint, face image). Usually, in order to accurately identify biological information, there is always a trade-off between recognition performance and security^58,59^. While in the Stokes meta-hologram, the complete decoupled modulation of amplitude, phase, and polarization can encrypt and reconstruct the secret images accurately compared with other holograms (e.g., phase-only and amplitude-only holograms), which ensures that biometric information can be stored in high security and identified accurately^60,61^. Here, the robustness performance is also analyzed, and it is proved that the Stokes meta-hologram has great anti-shearing and certain anti-noise capacities (detailed in Supplementary Note 8). Furthermore, the Stokes operators are the direct extension of their classical counterparts, Stokes meta-hologram may open a new avenue for encoding quantum information via polarization manipulation^62^.

To summarize, we proposed and demonstrated an optical encryption scheme, referred to as Stokes meta-hologram, to address the performance issues of optical cryptography. By integrating visual cryptography with a metasurface-assisted encoding technique, the secret images can be vectorially encrypted into unrecognizable visual patterns with spatially distributed polarization based on polarization pattern masking. In experimental verifications, we designed a dual-channel meta-hologram, which introduces polarization-dependent interference through a tetratomic macro-pixel arrangement to construct dual-channel far-field fully-polarized light fields. Hierarchical encryption strategies were exhibited, including Stokes vector encryption, Mueller matrix encryption, and angular vector encryption. Moreover, we presented an asymmetric encryption scheme based on Stokes vector rotation transformation to further boost security. The inherent invisible property of polarization, together with multichannel vectorial encryption, can effectively improve security and capacity. In addition, our scheme largely enriches the functionality breadth of metasurface-based optical cryptography, and could offer an unprecedented information security solution for data transfer and exchange in optical communication.

## Methods

### Fabrication

The metasurfaces were fabricated based on the process of deposition, patterning, and etching. At first, a 550-nm-thick Poly-Si film was deposited on a 500-μm-thick fused silica substrate by inductively coupled plasma enhanced chemical vapor deposition (ICPECVD), and then a 100-nm-thick Hydrogen silsesquioxane electron beam spin-on resist (HSQ, XR-1541) was spin-coated onto the Poly-Si film. Next, the desired structures were imprinted by using standard electron beam lithography (EBL, Nanobeam Limited, NB5) and subsequently developed in NMD-3 solution (concentration 2.38%) for 2 minutes. Finally, by using inductively coupled plasma etching (ICP, Oxford Instruments, Oxford Plasma Pro 100 Cobra300), the desired structures were transferred from resist to the Poly-Si film.

### Experimental characterization

The fabricated metasurfaces were illuminated by a laser beam at *λ* = 633 nm, which has uniform intensity and polarization state of |*H*〉. The transmitted light beam propagated freely (enough away from the metasurface) and its far-field was projected onto a white screen, then captured by a camera (Supplementary Fig. 11). In Stokes vector measurement, a linear polarizer and a cascade of a linear polarizer and quarter-wave plate were used to obtain the linear polarization components |*H*〉, |*V*〉, |*A*〉, |*D*〉, and circular polarization components |*R*〉, |*L*〉, respectively. Off-axis configuration was adopted to separate dual channels and avoid unmodulated components. The measured efficiencies of metasurfaces were about 60%, the loss mainly stemmed from the absorption of the material and imperfect fabrication, which caused the absorptivity and unmodulated components about 32 and 8% in our experiment.

## Supplementary information

Supplementary Information

## Data Availability

The data that support the findings of this study are available from the corresponding author upon request.
