# Supplementary material for: Stokes meta-hologram toward optical cryptography
Source: Nat Commun. 2022 Nov 5;13:6687. doi: 10.1038/s41467-022-34542-9 (PMC9637117; doi:10.1038/s41467-022-34542-9)
Supplement: Supplementary file 1 — Supplementary Information [file 41467_2022_34542_MOESM1_ESM.docx]

**Supporting Information for “Stokes meta-hologram toward optical cryptography”**

Xuyue Guo^1^, Peng Li^1*^, Jinzhan Zhong^1^, Dandan Wen^1^, Bingyan Wei^1^, Sheng Liu^1^, Shuxia Qi^1^, and Jianlin Zhao^1*^

^1^ *Key Laboratory of light field manipulation and information acquisition, Ministry of Industry and Information Technology, and Shaanxi Key Laboratory of Optical Information Technology, School of Physical Science and Technology, Northwestern Polytechnic**al University, Xi’an* *710129, China*

^*^*E-mail: pengli@nwpu.edu.cn; jlzhao@nwpu.edu.cn*

**Supplementary Note 1: Meta-hologram design**

Considering the powerful ability of metasurface for manipulating spin states, the desired fully-polarized light field is firstly decomposed under circular polarization eigenstates and can be expressed as

. (1)

Where, |*R*〉 and |*L*〉 represent the right- (RCP) and left-hand circular polarizations (LCP), and *A*_R_, *φ*_R_ and *A*_L_, *φ*_L_ are the amplitude and phase distributions of RCP and LCP, respectively.

Based on effective medium theory, each individual meta-atom behaves as a birefringent wave plate, which exhibits different amplitude and phase responses on the components polarized along its ordinary and extraordinary axes, respectively. The modulation effect can be described with Jones matrices as

. (2)

Where, *R*(*θ*) is the rotation matrix, (*T_o_*, *T_e_*) and (*φ_o_*, *φ_e_*) are the transmission amplitudes and phases along the ordinary and extraordinary axes, respectively, as shown in Fig. 2a. Generally, the transmitted amplitudes are considered uniform, i.e. *T_o_*=*T_e_*=*T*, therefore the Jones matrix can be simplified to

, (3)

where *δ*=(*φ_o_*−*φ_e_*) and *φ*_0_=(*φ**_o_*+*φ_e_*)/2 depict the phase retardation between ordinary and extraordinary components and propagation phase for circular polarization, respectively.

For circularly polarized incident beams, i.e., **E***_in_* = [1 ±i]^T^, the output fields can be derived as

. (4)

The output field naturally consists of two components with orthogonal polarizations, namely, the co- and cross-polarized components. When the meta-atom behaves as a half-wave plate, i.e., *δ*=π, the co-polarized component is eliminated. Therefore, for a linearly polarized incident beam, the output field can be expressed as

. (5)

Clearly, the two independent phase distributions *φ*_0_+2*θ* (*φ*^+^) and *φ*_0_−2*θ* (*φ*^−^) can be imposed on orthogonal circular polarizations. Because the geometric phase and the propagation phase are both needed, the meta-atom requires varying geometrical size and rotation angle, which can be related to the phase distribution as

. (6)

Further, to realize the simultaneously modulation of amplitude and phase, the polarization dependent interference is introduced through the tetratomic macro-pixel arrangement. Each macro-pixel unit-cell comprises four types of rectangular nanostructures denoted as meta-atoms *M*_1_, *M*_2_, *M*_3_, *M*_4_, which can be regarded as different interference sources. For linearly polarized light beam incidence, each meta-atom yields two components with orthogonal circular polarizations:

, (7)

where, |*R*〉 and |*L*〉 represent RCP and LCP, and *i*=*M*_1_, *M*_2_, *M*_3_, *M*_4_. The output components with the same polarization state will overlap and experience constructive or destructive interference to construct the dual-channel output fields:

. (8)

If we set the preestablished phase distributions as

$\begin{matrix} \varphi_{M_{1}}^{+}=\phi_{11}+\xi_{11},\varphi_{M_{1}}^{-}=\phi_{12}+\xi_{12} \\ \varphi_{M_{3}}^{+}=\phi_{11}-\xi_{11},\varphi_{M_{3}}^{-}=\phi_{12}-\xi_{12} \\ \varphi_{M_{2}}^{+}=\phi_{21}+\xi_{21},\varphi_{M_{2}}^{-}=\phi_{22}+\xi_{22} \\ \varphi_{M_{4}}^{+}=\phi_{21}-\xi_{21},\varphi_{M_{4}}^{-}=\phi_{22}-\xi_{22} \end{matrix}$, (9)

the dual-channel output fields thus can be described as

. (10)

As these parameters in Supplementary Eq. (9) are independent, the simultaneous and independent modulation of both amplitude and phase on orthogonal circular polarizations can be achieved. Consequently, complete decoupled modulation of amplitude, phase, and polarization can be realized to construct fully-polarized field in the far-field. Because of the complex amplitude modulation, the reconstructed light field has good reconstruction quality. In the experiment, we analyzed the two images reconstructed in Fig. 2d, the MSE (Mean Squared Error) of the reconstructed images in the two channels are 0.0478 and 0.0634, respectively

**Supplementary Note 2: Stokes vector encryption**

Two special types of information encryption are simultaneously exhibited in two channels, i.e., encrypting arbitrary three grayscale images and a RGB colour image into respective Stokes vectors (*S*_1_ *S*_2_ *S*_3_)^T^, as shown in Supplementary Fig. 1. In channel-1, three different grayscale images shown in Supplementary Fig. 1a are encrypted into three Stokes parameters *S*_1_, *S*_2_, *S*_3_ as secret images, respectively. To decrypt the information, we use intensity measurement method to determine the Stokes vector (*S*_1_ *S*_2_ *S*_3_)^T^ through Supplementary Eq. (3). Supplementary Fig. 1b shows the measured total intensity and the intensity distribution of each component. The decrypted information is shown in Supplementary Fig. 1c. In channel-2, the RGB components of a colour image (Supplementary Fig. 1d) are encrypted into *S*_1_, *S*_2_, and *S*_3_, respectively. The experiment results and decrypted colour image are shown in Supplementary Figs. 1e and 1f. The corresponding simulation results are shown in Supplementary Fig. 2.

**
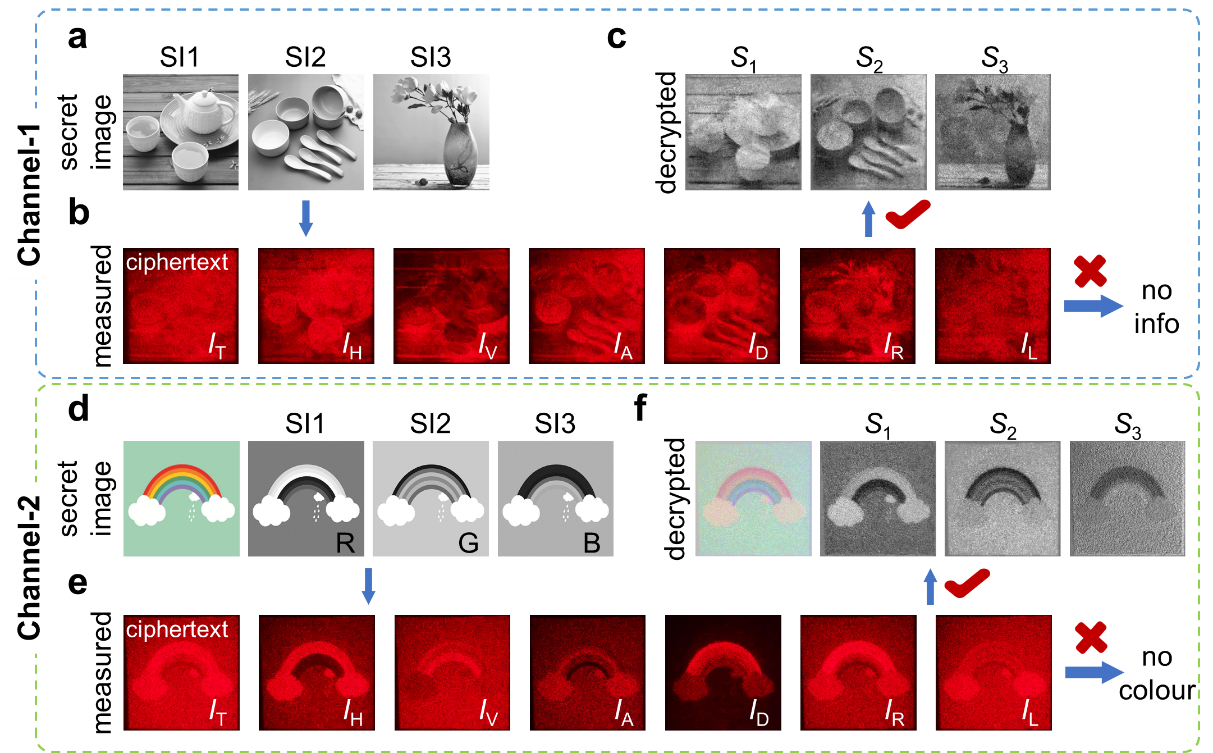
**

**Supplementary Fig. 1. Experiment results of Stokes vector encryption.** **a**, **d** Two types of information encryptions in two channels, which exemplify the encryptions of arbitrary three grayscale images (upper panel) and a RGB colour image (lower panel). SI: secret images. **b**, **e** The measured intensity distributions of different polarization components. **c**, **f** The decrypted secret images and recovered RGB image. It is obvious that no secret image can be directly acquired from direct polarization observation.


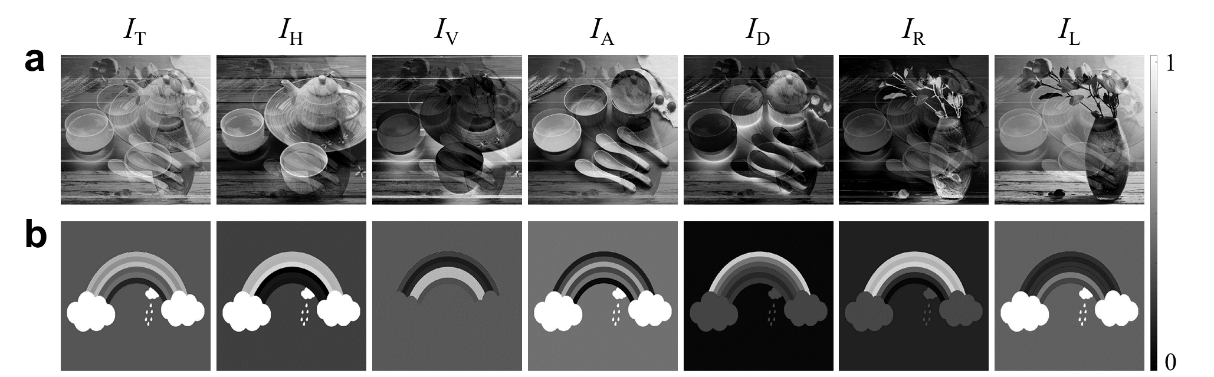


**Supplementary Fig. 2.** **Simulation results of Stokes vector encryption.** Simulation results of the ciphertext patterns (total intensity *I*_T_) and the intensity distributions of different polarization components in two channels with Stokes vector encryption.

**Supplementary Note 3: Mueller** **matrix encryption**

In Mueller matrix encryption, the Mueller matrix is chosen as a cascade of a quarter-wave plate and a half-wave plate, of which the characterical angle distributions of fast axes are described by 5×5 pixeled martrices. Supplementary Fig. 3 shows the process of Mueller matrix encryption, in which the secret images are mapped into Stokes vector (*S*_1_ *S*_2_ *S*_3_)^T^, and modulated by Mueller matrices *M*_1_ and *M*_2_. The middle panel of Supplementary Fig. 3 shows the intensity distributions of different polarization components, and the corresponding simulation results are shown in Supplementary Fig. 4. To decrypt, the measured Stokes vector need to be multiplied by the inverse of the Mueller matrix, i.e., [*M*_2_*M*_1_]^−1^.


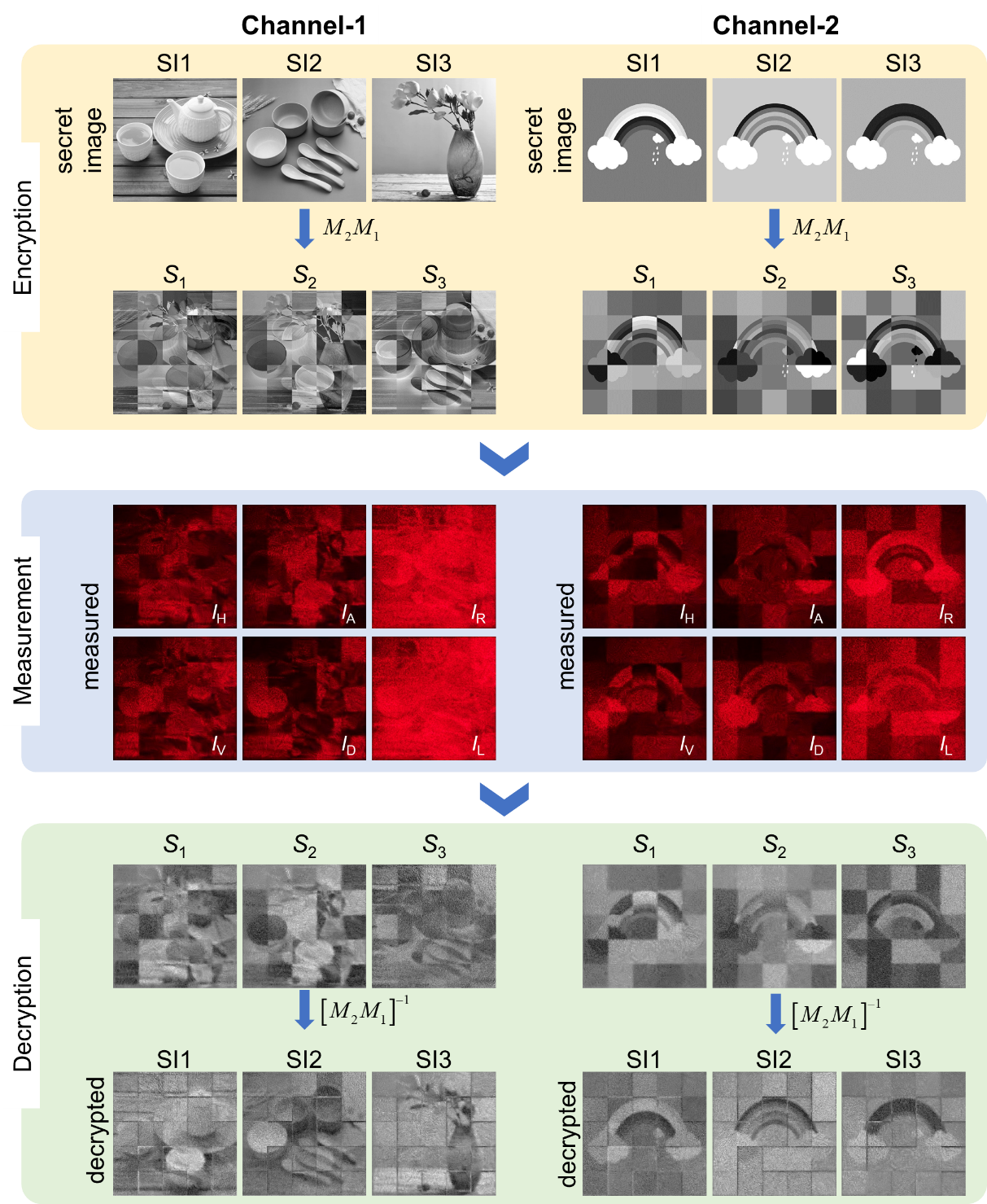


**Supplementary Fig. 3.** **Simulation and experiment results of Mueller matrix encryption process in different channels.** SI: secret images.


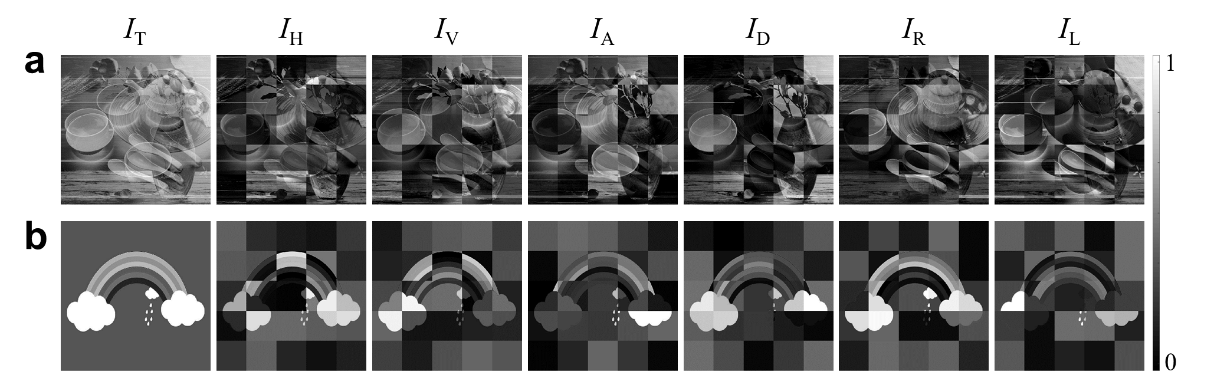


**Supplementary Fig. 4.** **Simulation results of Mueller matrix encryption.** Simulation results of the ciphertext patterns (total intensity *I*_T_) and the intensity distributions of different polarization components in two channels with Mueller matrix encryption.

**Supplementary Note 4: Angular vector encryption on Poincaré sphere**

**
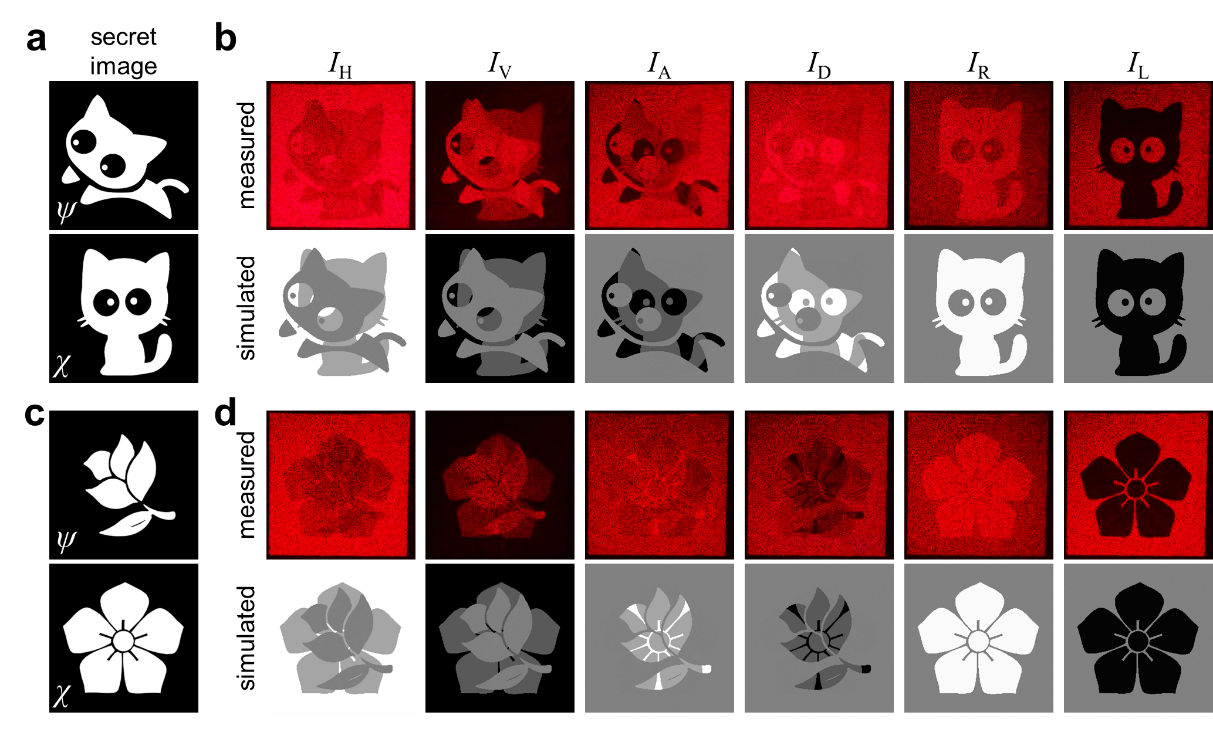
**

**Supplementary Fig. 5.** **Simulation and supplementary experiment results corresponding to the experiment results shown in Fig. 4a in the main text.** **a**, **c** Secret images for azimuth and elliptical angle encryption with uniform total intensity distribution. **b**, **d** Measured and simulated intensity distributions of different polarization components.

**
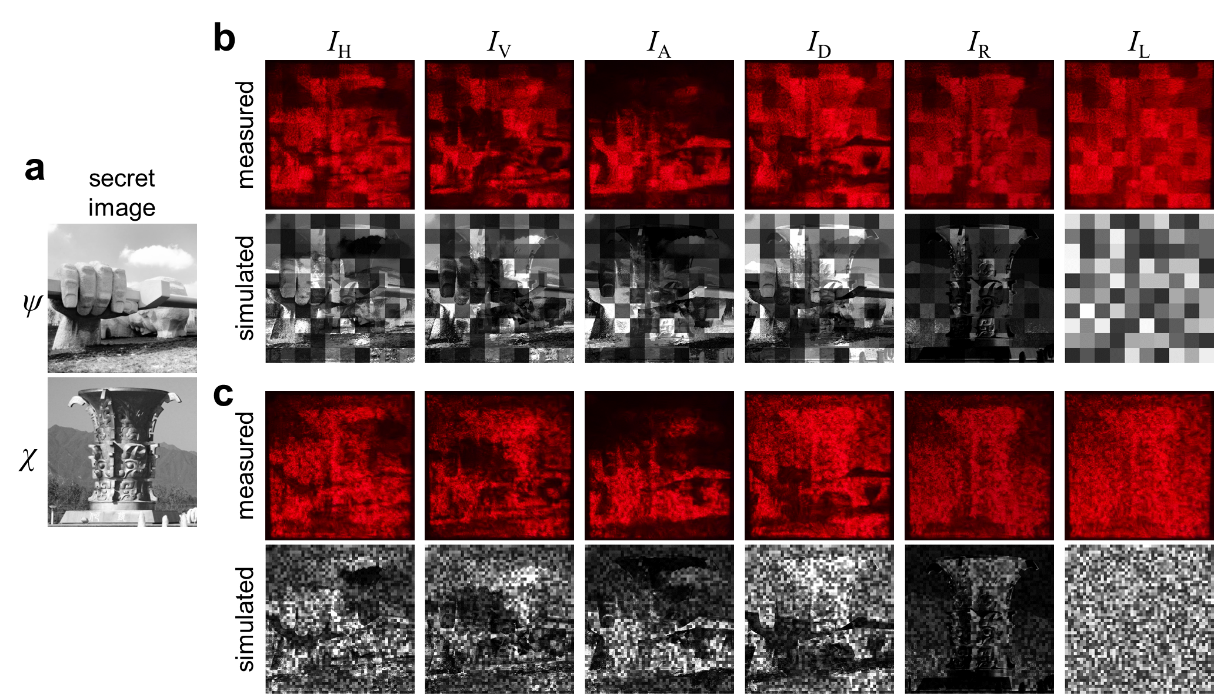
**

**Supplementary Fig. 6.** **Simulation and supplementary experiment results** **corresponding to the experiment results shown in Fig. 4b in the main text.** **a** Secret images for azimuth and elliptical angle encryption with ununiform total intensity distribution. **b,** **c** Measured and simulated intensity distributions of different polarization components. The random intensity distributions are spatially divided into 10×10 (**b**) and 50×50 (**c**), respectively. The two images are photographs of architectures on the campus of Northwestern Polytechnical University, and are used with permission of Northwestern Polytechnical University.

**
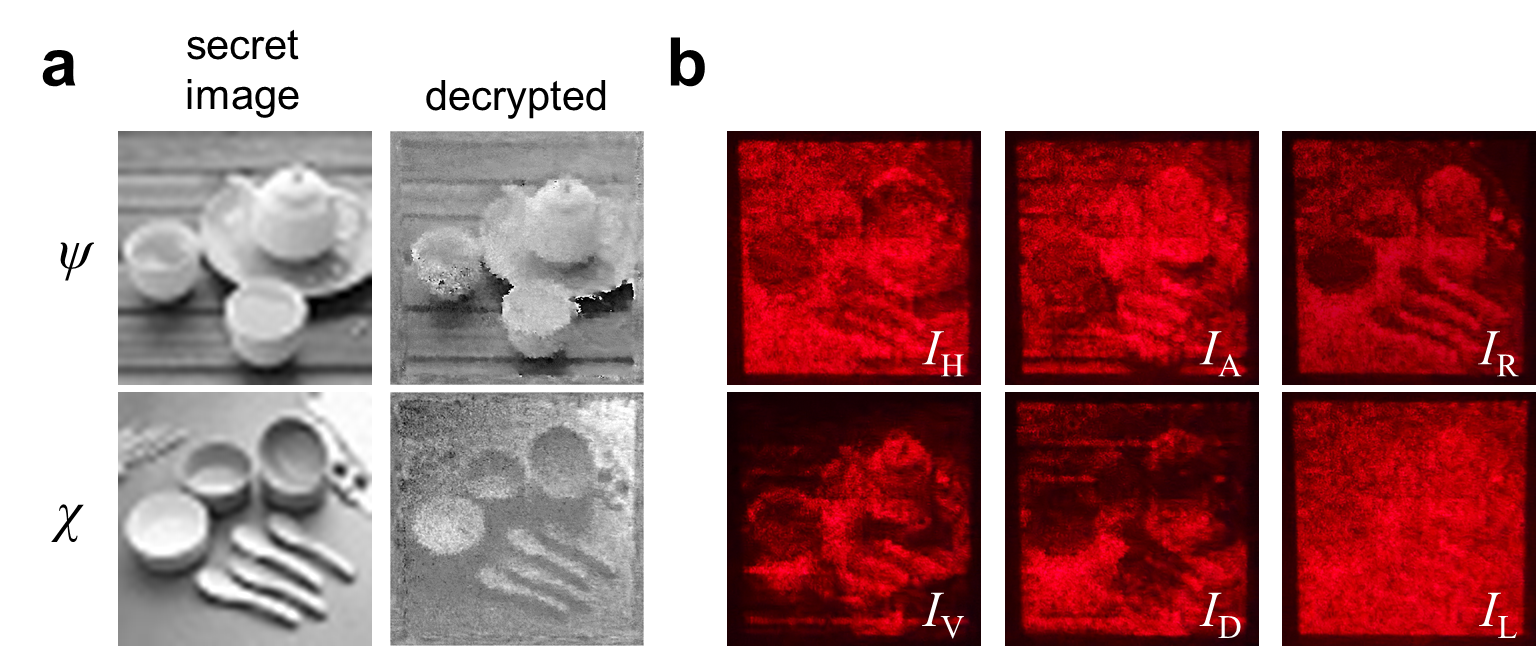
**

**Supplementary Fig. 7.** **Supplementary experiment results corresponding to the experiment results shown in Fig. 4b in the main text.** **a** Secret images and decrypted azimuth and elliptical angles. **b** Measured intensity distributions of different polarization components. The secret images have pixels of 50×50, and the random intensity distributions are spatially divided into 50×50 pixels.

**Supplementary Note 5: Asymmetric encryption**

Supplementary Fig. 8 shows the switch relationship between the normal and redefined Poincaré spheres under the |*H*〉-|*V*〉 and |*A*〉-|*D*〉 eigenstates. It can be seen directly from Supplementary Fig. 8 that the rotation relationships are

, (11)

where **ŝ**_1_, **ŝ**_2_, and **ŝ**_3_ stand for the unit vector **n** of the rotation axis, and *ϕ* depicts the rotation angle, which are obtained from the Jones matrix U:

, (12)

where,

$\sigma=\sigma_{1}\hat{s}_{1}+\sigma_{2}\hat{s}_{2}+\sigma_{3}\hat{s}_{3}$. (13)

In decryption process, due to the special rotation symmetry of Poincaré sphere, the measured Stokes vector **S**_m_=(*S*_m1_, *S*_m2_, *S*_m3_)^T^ needs undergoing an identical rotation process, i.e., **S**=R(U)**S**_m_, to decrypt the secret images masked in original Stokes vector **S**=(*S*_1_, *S*_2_, *S*_3_)^T^, the decryption process and the relationship between **S**_m_ and **S** can be expressed as

$\begin{matrix} |H\rangle\text{-}|V\rangle:\left(\begin{matrix} S_{1} \\ S_{2} \\ S_{3} \end{matrix}\right)=\left(\begin{matrix} 0 & 1 & 0 \\ 0 & 0 & 1 \\ 1 & 0 & 0 \end{matrix}\right)\left(\begin{matrix} S_{\text{m1}} \\ S_{\text{m2}} \\ S_{\text{m3}} \end{matrix}\right)=\left(\begin{matrix} S_{\text{m2}} \\ S_{\text{m3}} \\ S_{\text{m1}} \end{matrix}\right)\text{ }\to\text{ }\begin{matrix} S_{\text{m1}}=S_{3} \\ S_{\text{m2}}=S_{1} \\ S_{\text{m3}}=\text{S}_{2} \end{matrix} \\ |A\rangle\text{-}|D\rangle:\left(\begin{matrix} S_{1} \\ S_{2} \\ S_{3} \end{matrix}\right)=\left(\begin{matrix} -1 & 0 & 0 \\ 0 & 0 & 1 \\ 0 & 1 & 0 \end{matrix}\right)\left(\begin{matrix} S_{\text{m1}} \\ S_{\text{m2}} \\ S_{\text{m3}} \end{matrix}\right)=\left(\begin{matrix} -S_{\text{m1}} \\ S_{\text{m3}} \\ S_{\text{m2}} \end{matrix}\right)\text{ }\to\begin{matrix} \text{ }S_{\text{m1}}=-S_{1} \\ S_{\text{m2}}=S_{3} \\ S_{\text{m3}}=S_{2} \end{matrix} \end{matrix}$. (14)

Supplementary Figs. 9 and 10 show the simulation results of original Stokes vector (*S*_1_, *S*_2_, *S*_3_)^T^ and measured Stokes vector (*S*_m1_, *S*_m2_, *S*_m3_)^T^, which are consistent with the relationship obtained in Supplementary Eq. (14).


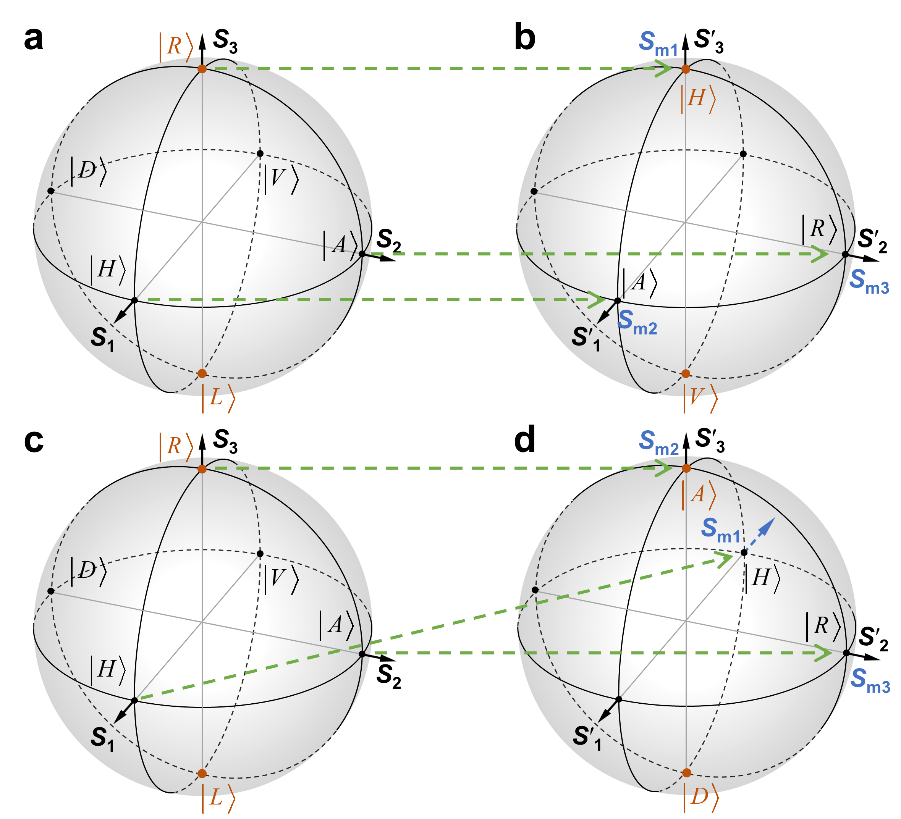


**Supplementary Fig. 8.** **Transformation relationship.** Transformation relationship between the normal (**a**, **c**) and redefined Poincaré sphere under the |*H*〉-|*V*〉 (**b**), |*A*〉-|*D*〉 (**d**) eigenstates.

**
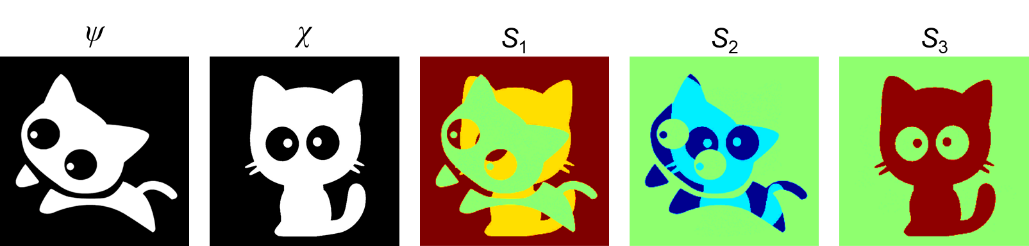
**

**Supplementary Fig. 9.** **Simulation results corresponding to the experiment results shown in Fig. 5 in the main text.** Secret images encrypted on azimuth and ellipticity and the original Stokes vector (*S*_1_, *S*_2_, *S*_3_)^T^ on the normal Poincaré sphere.


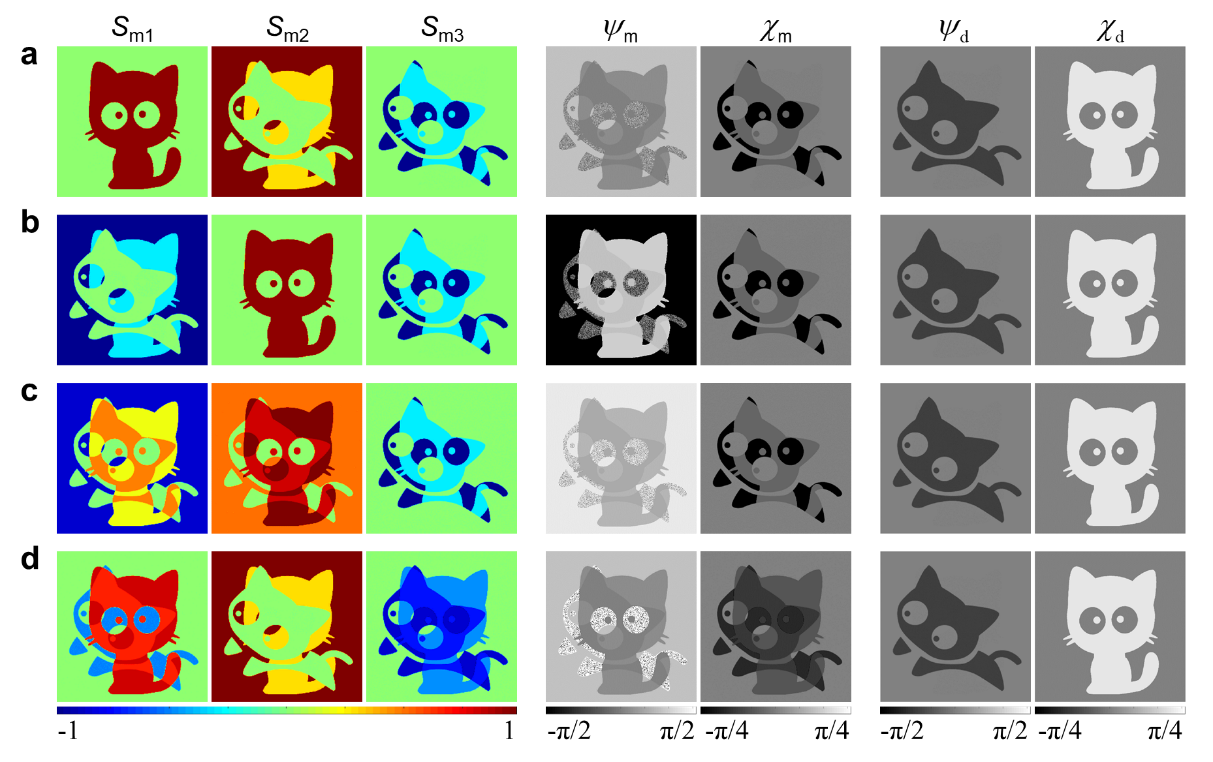


**Supplementary Fig. 10.** **Simulation results corresponding to the experiment results shown in Fig. 5 in the main text.** Simulation results of Stokes vector (*S*_m1_ *S*_m2_ *S*_m3_)^T^, directly decrypted azimuth (*ψ*_m_) and elliptical angles (*χ*_m_) based on normal Poincaré sphere, as well as correctly decrypted azimuth (*ψ*_d_,) and elliptical angles (*χ*_d_) based on the rotational Poincaré sphere. **a** |*H*〉-|*V*〉. **b** |*A*〉-|*D*〉. **c** |*α*_1_〉-|*β*_1_〉. **d** |*α*_2_〉-|*β*_2_〉. The coordinates of |*α*_1_〉 and |*α*_2_〉 are (π/3, π/2) and (0, π/3), respectively. Corresponding to the experiment results shown in Figs. 5**b**-**d** in the main text.

**Supplementary Note 6: Experimental setup**


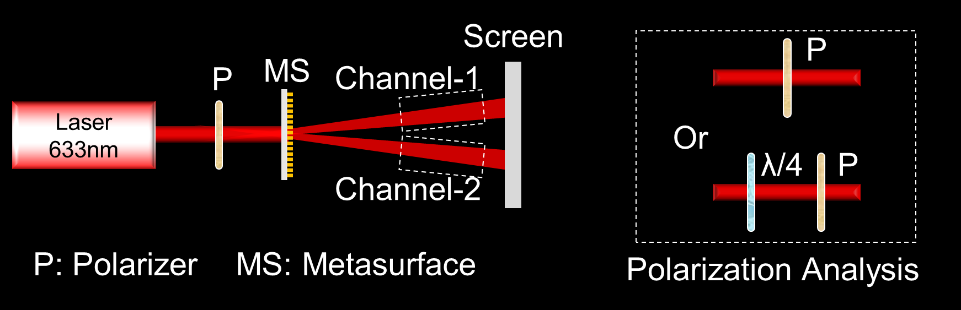


**Supplementary Fig. 11.** **Experimental setup.** The MS is illuminated by a 633nm laser beam with polarization state of |*H*〉 after a polarizer (P). The transmitted light beam propagates freely and projects onto a white screen, and the intensity distributions are captured by a camera. The white frame shows the polarization analysis setup for obtaining the linear polarization components (upper), and circular polarization components (lower).

**Supplementary Note 7: Security analysis**

In cryptanalysis, Kerckhofs’s principle^1^ assumes that the attacker knows the cryptosystem being used, and the ordinarily used attack methods mainly include ciphertext only attack (COA)^2-5^, known-plaintext attack (KPA)^2, 3, 6, 7^, and chosen-plaintext attack (CPA)^2, 5^. The COA cracks the secret information by brute force attack and testing all secret key combinations. The KPA assumes that the attacker knows several ciphertext-plaintext pairs to crack the secret key and encryption algorithm, and the CPA assumes that the attacker knows the encryption algorithm (any ciphertext-plaintext pair can be obtained) to crack the secret key.

To evaluate the security performance of proposed Stokes meta-holograms, it should be firstly noted that, although the ciphertexts are generated from complex-amplitude hologram, but the secret images are encrypted into the polarization after polarization pattern masking (Stokes vector, Mueller matrix, angular vector and eigenstate transformation), therefore the Stokes meta-hologram is immune to phase-retrieval-based KPA. Meanwhile, the accessible ciphertext-plaintext pairs are random (associated with the Mueller matrix, random intensities) and plaintext-dependent, which makes it difficult to build a database for training and the neural-network- or the deep-learning- based attack^8^ is difficult to carry out. Therefore, here we focus on cracking the polarization mask.

For the fundamental level encryption, i.e., Stokes vector encryption, here we assume that the attacker already knows that the secret images are encrypted in polarization. Then for the cracking ciphertext (*I*_T_ in Supplementary Fig. 1) with COA, the attacker needs to test every possible polarization state through polarization analysis, of which the simulation results are shown in Supplementary Fig. 12. Clearly, the secret images cannot be cracked directly by single polarization analysis, which improves the security to a certain extent compared with previous works^9, 10^. However, the intensity distribution of individual component gives away some information, leaving a possibility for attacker to recognize the secret images, and through KPA, the attacker can perform further operations on multiple images that have been detected. Therefore, the fundamental level encryption still has a large risk of being cracked, so we further introduce polarization mask to improve the security.


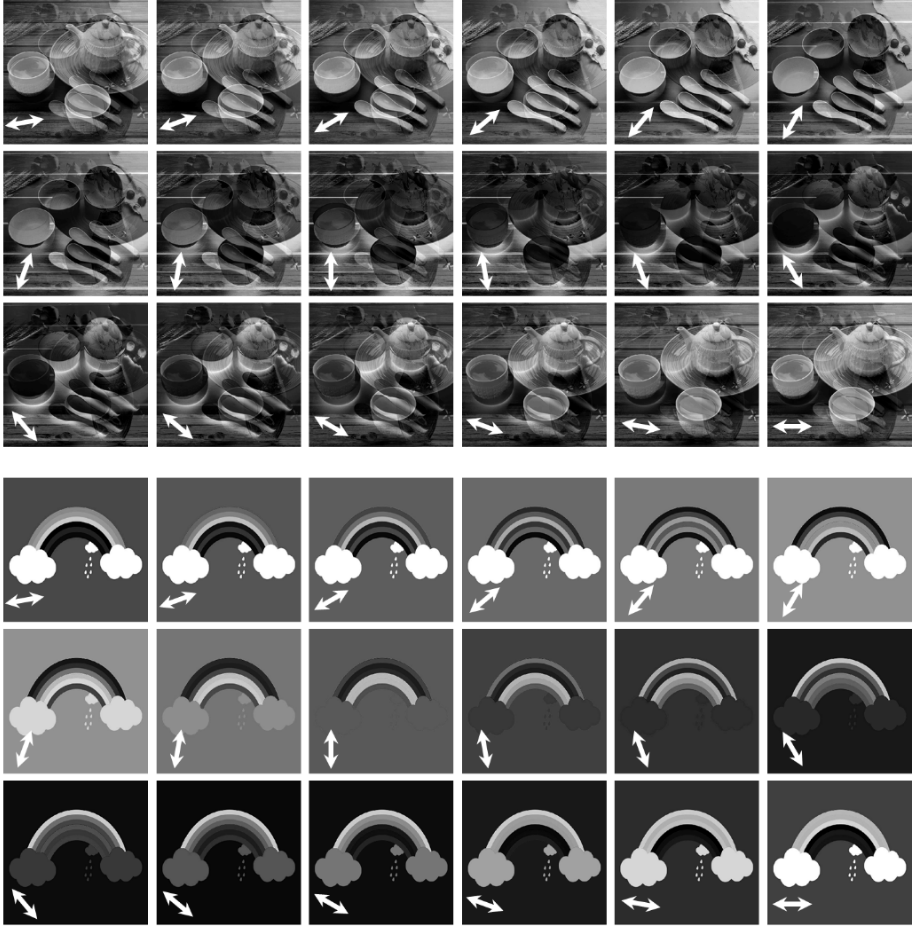


**Supplementary Fig. 12.** **Simulation results of linear polarization analysis for Stokes vector encryption.** The white arrows indicate the direction of the polarization analyzer. Corresponding to the ciphertext in Supplementary Fig. 1.

For Mueller matrix encryption, we assume that the attacker already knows that the secret images are encrypted in Stokes vector (encryption algorithm) with a polarization mask. At this point, simple polarization analysis (Supplementary Fig. 13a) doesn't work except cracking the Mueller matrix, and considering the pixelated distribution of modulation type is almost impossible to leak, the secret key can be divided to three parts: the cascade number (*C*_num_), the cascade order (*C*_order_), the modulation type (*C*_type_). For COA, Supplementary Figs. 13b and 13c show the simulation results of randomly generating the above three factors and attacking the ciphertext for 1000 times. Here, we adopt average normalized mean-squared error (NMSE, the average value of three images’ NMSE) to evaluate the recovered images. Clearly, the most of the NMSE values are between 2 and 10, indicating that the gray value of the recovered image is many times worse than the original image, since such value of right decrypted image is almost zero.

For KPA, three cases are considered: (1) known *C*_num_ and *C*_order_, unknown *C*_type_; (2) known *C*_num_ and *C*_type_, unknown *C*_order_, (3) known *C*_num_, *C*_type_ and *C*_order_, and the simulated average NMSEs are shown in Supplementary Fig. 14. It can be seen that there are no particularly large values and the NMSE are concentrated around 2, indicating that KPA is easier than COA, but such NMSE value is still not enough to crack the secret images. Meanwhile, even if attacker knows the encryption algorithm but don't know the pixelated distribution, the CPA can only be done in a similar way as above, and the unavailability of 1000 times random attacks shows that the Mueller matrix encryption is also secure against CPA.


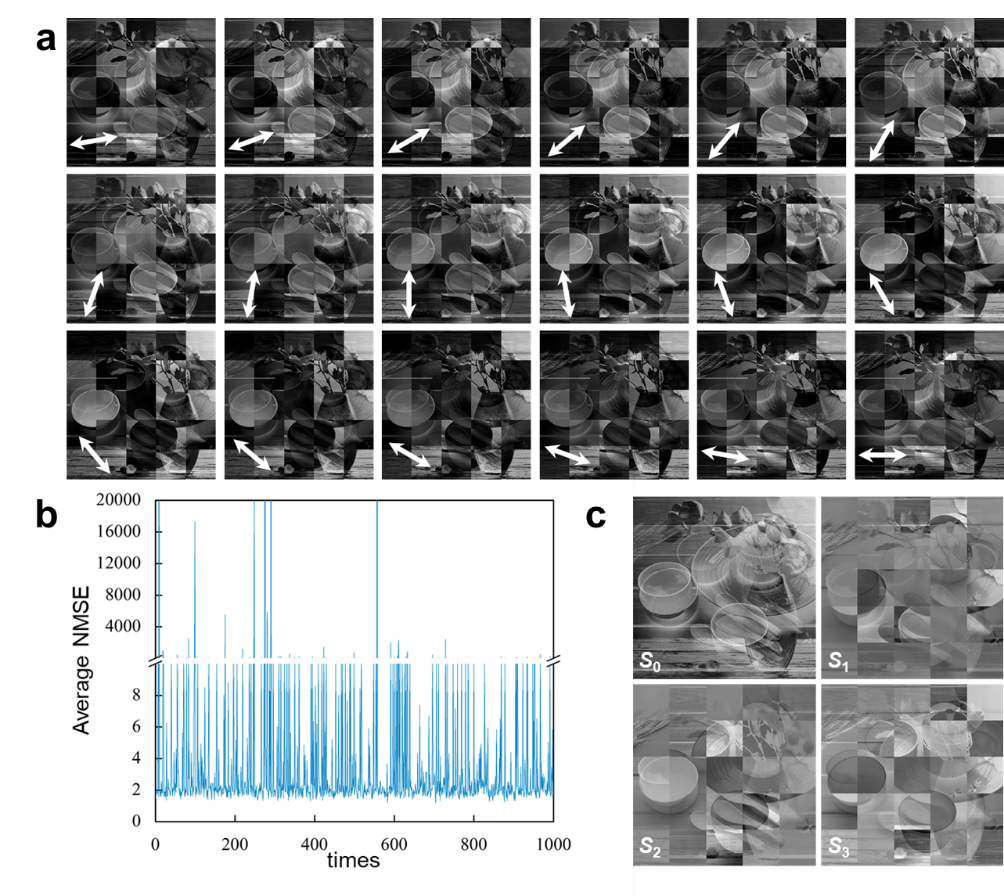


**Supplementary Fig. 13.** **Simulation results of COA for Mueller matrix encryption.** **a** Simulation results of linear polarization analysis, corresponding to the ciphertext in Fig. 3b. The white arrows indicate the direction of the polarization analyzer. **b** Average NMSE values distribution under 1000 times attack. **c** Ciphertext and decrypted images from one of 1000 attacks.


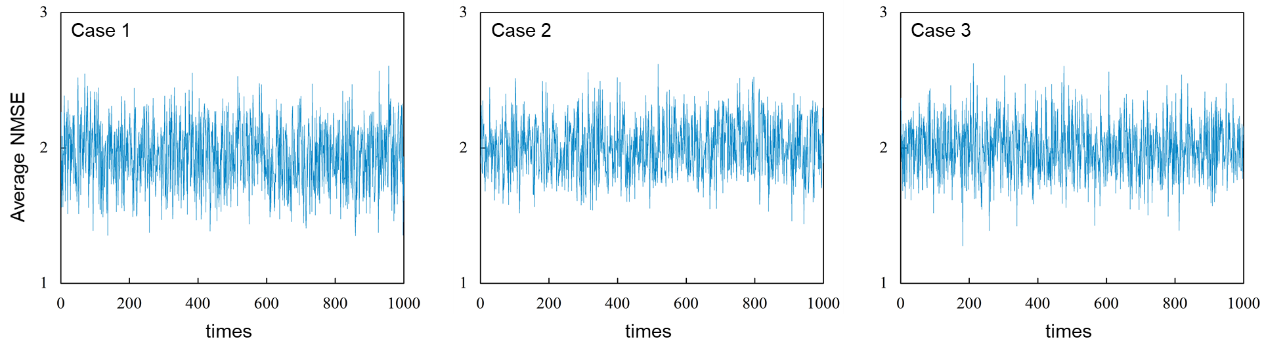


**Supplementary Fig. 14.** **Simulation results of KPA for Mueller matrix encryption.** Average NMSE values distribution under 1000 times attack of different cases.

Besides, it's worth pointing out that, the security level can be enhanced with more pixels. By increasing the pixel number, the randomness of encryption process can be increased effectively, and the complexity and the security of secret keys can be improved. Supplementary Fig. 15a shows the Stokes vector distributions of Mueller matrix encryption with 20×20, 50×50 and 750×750 pixels, and Supplementary Fig. 15b shows the simulated polarization analysis results corresponding to the case of 750×750 pixels. It can be seen that the more pixel number, the less information will be exposed by (*S*_1_, *S*_2_, *S*_3_)^T^ and individual component, which is more difficult to crack by KPA and CPA methods.


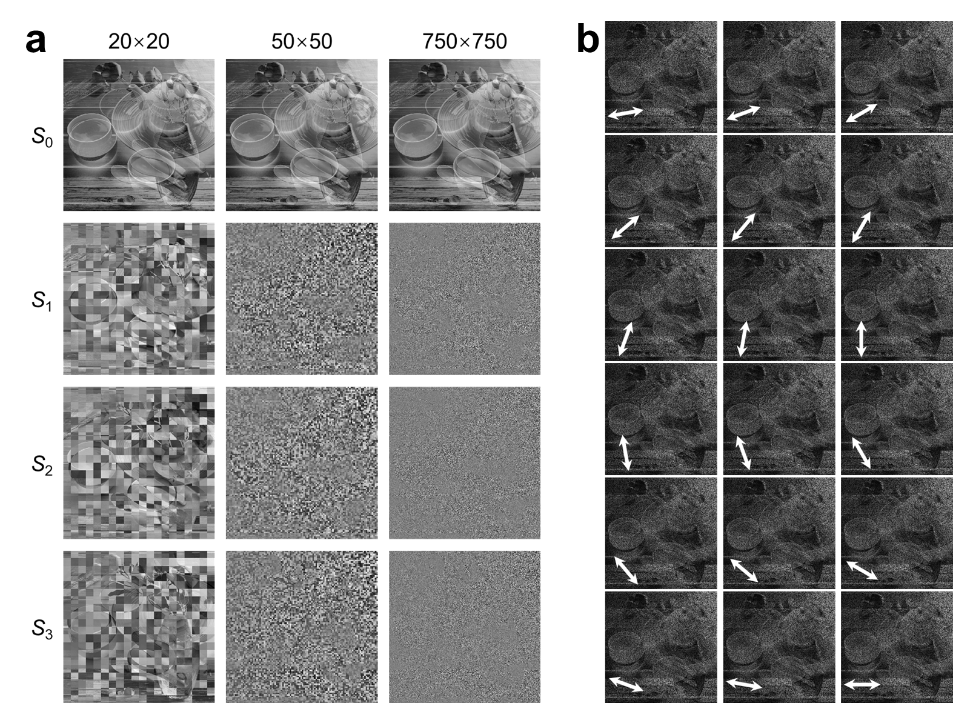


**Supplementary Fig. 15.** **Simulation results of linear polarization analysis for Mueller matrix encryption** **with more pixels. a** Stokes vector distributions of Mueller matrix encryption with 20×20, 50×50 and 750×750 pixels. **b** Simulation results of linear polarization analysis, corresponding to the ciphertext in **a**.

Obviously, the Mueller matrix has no modulation effect on the ciphertext (see *S*_0_ in Supplementary Fig. 15a), thus we extend the information carrier to angular vector to improve security. The random intensities attached to the ciphertext can efficiently dilute the information that can be directly observed, and similarly, the security level can also be enhanced with more pixels. Supplementary Fig. 16 shows an angular vector encryption with a 100×100 pixels of random intensities, and a Mueller matrix with 100×100 pixels is introduced. Clearly, no information can be obtained neither from the ciphertext (*S*_0_) nor the intensity distribution of any polarization components (*S*_1_, *S*_2_, *S*_3_, *I*_H_, *I*_V_, *I*_A_, *I*_D_, *I*_R_, *I*_L_, and polarization analysis results), whereas the secret images (*ψ*, *χ*) can be intactly decrypted by secret key. Besides, it is worth noting that, the introduced random intensity distribution can effectively improve the security of ciphertext from evaluation metrics of histogram analysis, correlation coefficients, and diﬀerential measures^11^.


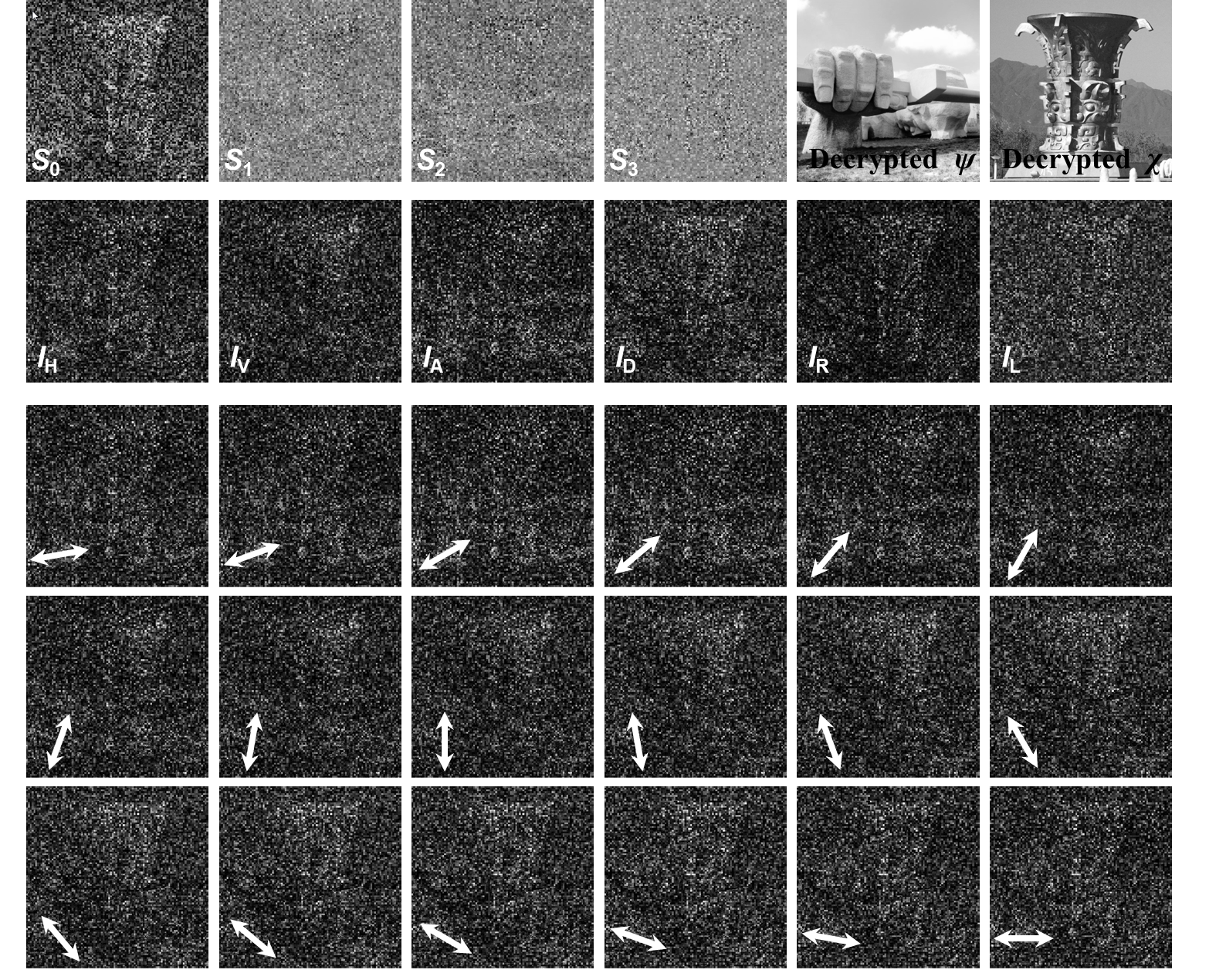


**Supplementary Fig. 16.** **Simulation results of linear polarization analysis for angular vector encryption with more pixels.** Simulation results of angular vector encryption with a 100×100 pixels of random intensities and Mueller matrix. The white arrows indicate the direction of the polarization analyzer. The two images are photographs of architectures on the campus of Northwestern Polytechnical University, and are used with permission of Northwestern Polytechnical University.

For asymmetric encryption scheme, the greatest security assurance is the asymmetry between the public and private keys. Although the public key is disclosed, as long as the private key is not compromised, the attacker can only crack it through COA. Supplementary Fig. 17 gives the average NMSE value distribution under 1000 times brute force attack. Clearly, the average NMSEs are concentrating around 4, indicating that asymmetric encryption has a certain increase in the difficulty of cracking. Same as above, Mueller matrices can also be introduced here to eliminate visible information in components.


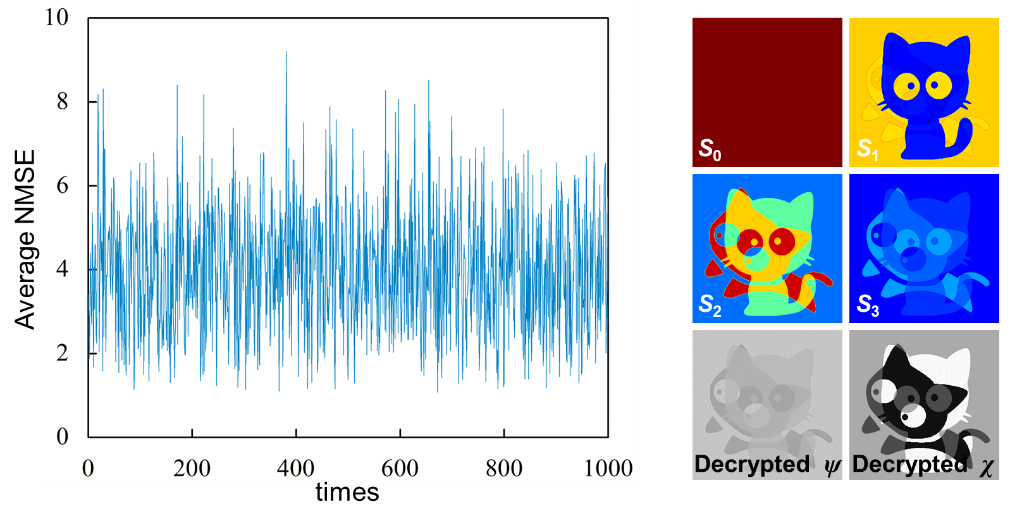


**Supplementary Fig. 17.** **Simulation results of COA for asymmetric encryption scheme.** Average NMSE values under 1000 times attack and ciphertext and decrypted images from one of 1000 attacks.

**Supplementary Note 8:** **Robustness** **analysis**

To assess the impact of defects introduced by fabrication and measurement, here numerical simulation of Mueller matrix encryption is adopted to examine the robustness of proposed Stokes meta-hologram, including anti-shearing and anti-noise capabilities.

First, the meta-device might be damaged during storage or transportation processes, and some of the information in the hologram will be lost. Therefore, we multiply the hologram by different binary filters (the first row of Supplementary Fig. 18a) to evaluate the anti-shearing capability, of which the decrypted images and their NMSE are shown in 18a. Thanks to the holographic method, the ciphertext is generated in the far-field, so the damage of meta-hologram will not have a great impact on the ciphertext, and the decrypted images are only different in gray distribution, while the entire features of secret images will be retained.

To evaluate anti-noise capability, we add a noise term on the ciphertext as *kN*, where *k* is noise coefficient, and *N* is random noise. The different noise distribution and corresponding decrypted images are shown in Supplementary Fig. 18b, which show that the secret images can still be decrypted under the influence of noise, but the feature information will be lost with the increase of noise.

Clearly, such anti-shearing and anti-noise capabilities is not available on nanoprint-based encryption scheme. For example, if the meta-device is damaged or under noise attack, part of the QR code is missing, making it difficult to get the cover image^12^, or the watermark image is directly damaged, thus losing the anticounterfeiting effect^13^.


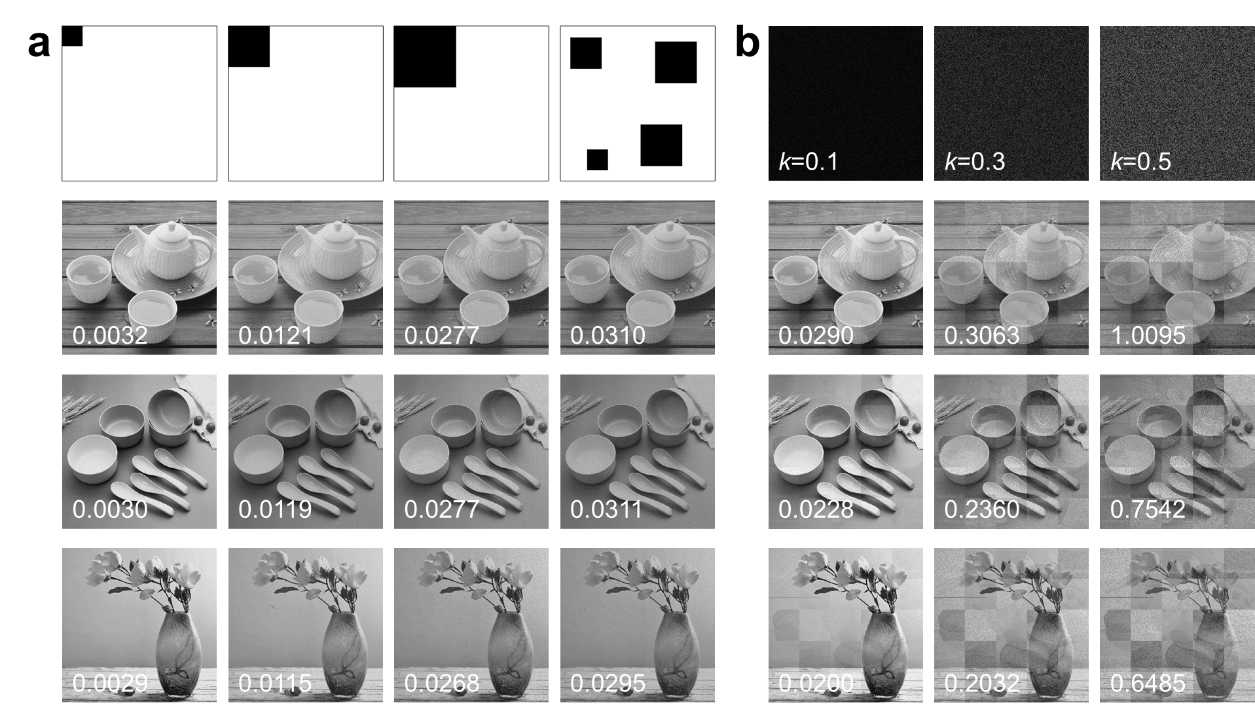


**Supplementary Fig. 18.** **Robustness analysis.** Simulation results of evaluating the (**a**) anti-shearing capability and (**b**) anti-noise capability. The number in the lower left corner is NMSE.

**References**

1. W. Stallings, *Cryptography and network security* (Prentice Hall, Englewood Clifs, NJ, 2004).

2. M. Dubreuil, A. Alfalou, and C. Brosseau. Robustness against attacks of dual polarization encryption using the Stokes–Mueller formalism. *J. Opt.* **14**, 094004 (2012).

3. S. Jiao, T. Lei, Y. Gao, Z. Xie, and X. Yuan. Known-plaintext attack and ciphertext-only attack for encrypted single-pixel imaging. *IEEE Access* **7**, 119557-119565 (2019).

4. G. Li, W. Yang, D. Li, and G. Situ. Cyphertext-only attack on the double random-phase encryption: experimental demonstration. *Opt. Express* **25**, 8690-8697 (2017).

5. Y. Frauel, A. Castro, T. J. Naughton, and B. Javidi. Resistance of the double random phase encryption against various attacks. *Opt. Express* **15**, 10253-10265 (2007).

6. X. Peng, P. Zhang, H. Wei, and B. Yu. Known-plaintext attack on optical encryption based on double random phase keys. *Opt. Lett.* **31**, 1044-1046 (2006).

7. J. Hou and G. Situ. Image encryption using spatial nonlinear optics. *eLight* **2**, 3 (2022).

8. M. Liao, S. Zheng, S. Pan, D. Lu, W. He, G. Situ, and X. Peng. Deep-learning-based ciphertext-only attack on optical double random phase encryption. *Opto-Electron. Adv.* **4**, 05200016 (2021).

9. Z. Deng, Q. Tu, Y. Wang, Z. Wang, T. Shi, Z. Feng, X. C. Qiao, G. P. Wang, S. Xiao, and X. Li. Vectorial compound metapixels for arbitrary nonorthogonal polarization steganography. *Adv. Mater.* **33**, 2103472 (2021).

10. R. Zhao, B. Sain, Q. Wei, C. Tang, X. Li, T. Weiss, L. Huang, Y. Wang, and T. Zentgraf. Multichannel vectorial holographic display and encryption. *Light:Sci. Appl.* **7**, 95 (2018).

11. C. M. DhiyaEddine, H. Rachid, A. Alfalou, H. Abderezzaq, and B. Badr-Eddine. Tailored dual polarization encryption-coherence modulation-based decryption scheme for a predefined uniformly distributed noisy output image. *Opt. Express* **30**, 17400-17415 (2022).

12. P. Zheng, Q. Dai, Z. Li, Z. Ye, J. Xiong, H. Liu, G. Zheng, and S. Zhang. Metasurface-based key for computational imaging encryption. *Sci. Adv.* **7**, eabg0363 (2021).

13. J. Deng, L. Deng, Z. Guan, J. Tao, G. Li, Z. Li, Z. Li, S. Yu, and G. Zheng. Multiplexed anticounterfeiting meta-image displays with single-sized nanostructures. *Nano Lett.* **20**, 1830-1838 (2020).
